# From nutrition to therapeutics: the diverse inflammopharmacological and biomedical roles of astaxanthin

**DOI:** 10.1007/s10787-025-02090-5

**Published:** 2026-01-09

**Authors:** Heba R. Ghaiad, Riham A. El-Shiekh, Ahmed M. Atwa, Aya M. Mustafa, Ali M. Elgindy, Mahmoud Abdelrahman Alkabbani, Weam A. Elkady, Kawther Magdy Ibrahim

**Affiliations:** 1 Department of Biochemistry, Faculty of Pharmacy, Cairo University, Cairo, 11562 Egypt; 2 Department of Pharmacognosy, Faculty of Pharmacy, Cairo University, Cairo, 11562 Egypt; 3 Department of Pharmacology and Toxicology, College of Pharmacy, Al-Ayen Iraqi University, AUIQ, An Nasiriyah, Iraq; 4 Department of Pharmacology and Toxicology, Faculty of Pharmacy, Egyptian Russian University, Cairo, Egypt

**Keywords:** Astaxanthin, Antioxidant, Anti-inflammatory, Bioactive compound, Disease prevention, Nutraceutical

## Abstract

Astaxanthin, a xanthophyll carotenoid derived primarily from *Hematococcus lacustris*, has been proposed as a potent bioactive compound demonstrating wide therapeutic applicability. In addition to its distinct molecular structure, astaxanthin has exceptional antioxidant property, surpassing that of other carotenoids and conventional antioxidants, while also exerting robust anti-inflammatory effects. The present review focuses on the current evidence of the complex multifaceted therapeutic actions of astaxanthin, including cardiovascular protection, neuroprotection, hepatoprotection, renal support, dermatological health, immune modulation, and emerging roles in metabolic disorders, reproductive health, and cancer prevention. Mechanistic insights highlight its potential to control key molecular mechanisms, including the NF-κB, Nrf2, MAPK, and TGF-β/Smad pathways, alongside the enhancement of endogenous antioxidant defenses. Preclinical and clinical findings have demonstrated benefits in conditions such as atherosclerosis, myocardial ischemia, nonalcoholic fatty liver disease, hypertension, Alzheimer’s disease, Parkinson’s disease, and inflammatory skin diseases. By integrating evidence drawn from molecular, experimental, and clinical studies, this review underscores astaxanthin’s potential as a complementary therapeutic agent and functional nutraceutical. The breadth of its bioactivity positions astaxanthin as a promising natural compound for targeted disease prevention and health promotion.

## Introduction

Natural products and compounds are vital for human health, offering unique pharmacological actions that contribute to the prevention and treatment of various diseases including cancers, inflammatory, cardiovascular, neurodegenerative, metabolic, and infectious diseases (Allam et al. 2025; Attallah et al. 2025; Marcus et al. 2025; Sadek et al. 2025; Salman et al. 2025). Astaxanthin (C_40_H_52_O_4_) is a lipophilic, dark reddish keto-carotenoid compound found in marine crustaceans, including shrimp and crabs, as well as in various microorganisms (Capelli et al. 2019). Even though this lipid-soluble compound lacks pro-vitamin A activity in humans, several previous reports have suggested that astaxanthin exhibits greater biological activity than many other carotenoids. Astaxanthin ranks among the most powerful antioxidants, with an activity reported to exceed that of α-tocopherol by a 100 time (Park et al. 2018). In fact, astaxanthin is classified as a natural food coloring agent by the European Commission (Authority 2005). Astaxanthin obtained from *Hematococcus lacustris* (formerly *H. pluvialis*, Chlorophyta) represents the major source for nutritional supplementation in humans and animals (Kidd 2011). Naturally, all carotenoids share a fundamental isoprene structure, characterized by a 40-carbon polyene backbone (Li et al. 2020a, b). The administration of astaxanthin can help in perverting or reducing the incidence of a variety of human and animal pathologies (Hussein et al. 2006; Yuan et al. 2011; Dhankhar et al. 2012; Yang et al. 2013). The potent antioxidative power of astaxanthin can be attributed to the ability of the ending ring part to trap free radicals. In 1991, astaxanthin was approved to be used as a dietary supplement because of its antioxidative potential (Chang and Xiong 2020). In this review, we comprehensively highlighted the bioactivities and health advantages of astaxanthin for disease prevention.

While numerous reviews have focused on the antioxidant and anti-inflammatory actions of astaxanthin, the present review uniquely provides a comprehensive and up-to-date collection of its multifaceted biological activities across all major body systems, supported by recent research. This review explores the actions of astaxanthin on cardiovascular health (e.g., atherosclerosis, hypertension, heart failure), neurological function (e.g., Alzheimer’s disease (AD), Parkinson’s disease (PD), depression), the hepatic and renal systems (e.g., nonalcoholic fatty liver disease, liver fibrosis, diabetic nephropathy), dermatological health, immunological modulation (e.g., cytokine regulation, immune cell activation), female reproductive health (e.g., PCOS, assisted reproductive outcomes), and auditory health (e.g., ototoxicity, hearing loss). Additionally, it has emerging applications in areas such as cancer therapy (e.g., apoptosis induction, tumor suppression), metabolic syndrome (e.g., lipid metabolism, insulin sensitivity), and ocular health (e.g., age-related macular degeneration, dry eye syndrome). By organizing the manuscript by body systems and detailing astaxanthin’s mechanistic actions, this review offers a holistic perspective on its therapeutic potential, bridging the gap between pre-clinical experimental evidence and therapeutic applications.

This review aimed to provide a mechanistic understanding of astaxanthin’s most relevant biological mechanisms and therapeutic targets emphasizing its antioxidant, anti-inflammatory, and mitochondrial regulatory actions. Moreover, particular attention was directed towards the effects of astaxanthin on key organ systems, such as cardiovascular, hepatic, renal, and nervous systems, where astaxanthin has demonstrated consistent experimental and clinical relevance.

## Phytochemistry

### Chemical structure

**Fig. 1:**
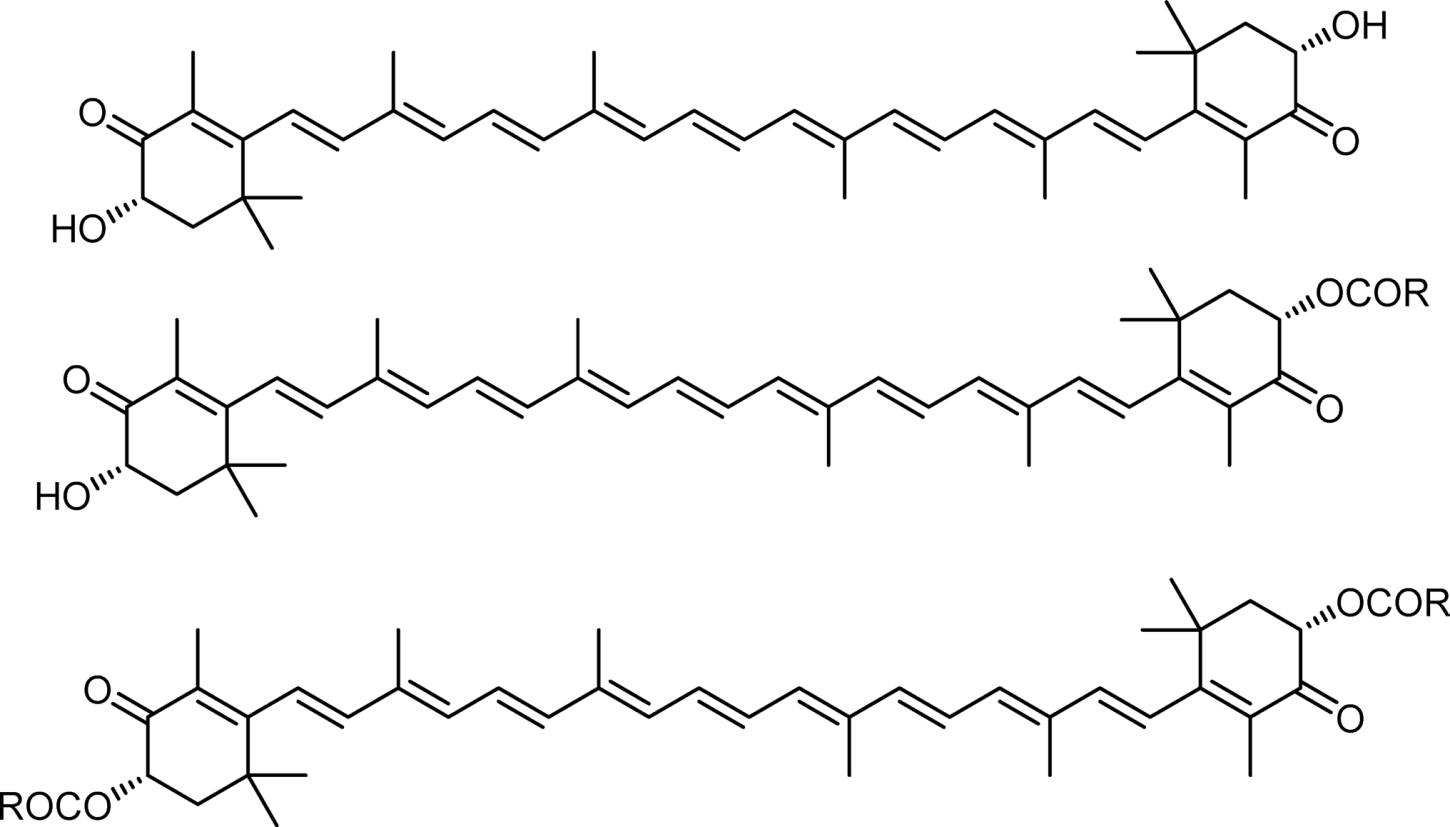
Structures of free astaxanthin, astaxanthin monoesters and astaxanthin diesters

Carotenoids can be broadly categorized into carotenes, composed solely of carbon and hydrogen, and xanthophylls, which represent their oxygenated derivatives. In xanthophylls, oxygen atoma may be present in the form of hydroxyl groups (e.g., zeaxanthin), or keto groups (e.g., canthaxanthin), or as a combination of both (e.g., astaxanthin). Astaxanthin (3,3′-dihydroxy-*β,β*-1-carotene-4,4′-dione) is composed of four isoprene units (Fig. 1) that are joined by conjugated double bonds to 2 terminal β-ionone rings. The hydroxyl group makes it possible for astaxanthin to be esterified, which augment its solubility and enables it to bond with fatty acids to create astaxanthin diesters and astaxanthin monoesters (Fig. 1). Esterification of one hydroxyl group with a fatty acid produces a monoester, while esterification of both generates a diester; such modifications enhance astaxanthin’s chemical stability and modulate its absorption and distribution in vivo. Unesterified astaxanthin molecules can occasionally be found in complexes with other macromolecules and in free form. Astaxanthin occurs as stereoisomers, geometric isomers, and in both free and esterified forms, all of which are naturally present in its biological sources. Owing to their molecular structure and interactions, the **esterified** forms have an antioxidant nature and are more chemically stable than the free forms. The stability of the esterified form is enhanced by a longer fatty acid chain, more esterification, and less fatty acid unsaturation. The most common natural stereoisomers are (3*S*, 3′*S*) and (3*R*, 3′*R*). The isomers (3*S*, 3′*S*), (3*R*, 3′*S*), and (3*R*, 3′*R*) make up synthetic astaxanthin. In plants, carotenoids are mostly found in *E*-configuration **geometric** isomers. Since all-E isomer are thermodynamically more stable, Z-isomers of astaxanthin are converted into this form during long-term storage. Notably, some Z-isomers demonstrate enhanced bioactivity, such as greater antioxidant, anti-inflammatory, and anti-aging properties, relative to E-isomers. The chemical formula for astaxanthin is C_40_H_52_O_4_. It has a molar mass of 596.84 g/mol (Al-Dbagh and Jwad 2020).

### Natural sources

Astaxanthin can be naturally found in algae, yeast, salmon, trout, krill, prawns and crayfish. Moreover, *Hematococcus lacustris* is an excellent source of natural astaxanthin. In wild and farmed Atlantic salmon (*Salmo salar*), astaxanthin concentrations range from 3–10 mg to 1–9 mg per each one kg of flesh, respectively. According to Iwamoto et al., taking 3.6 mg of astaxanthin per day can benefit one’s health (Iwamoto et al. 2000). Additionally, an earlier report explored the effects of dietary astaxanthin on stress resistance in *Pelteobagrus fulvidraco* or the yellow catfish. Supplementation with 80 mg/kg feed for 2 months has been demonstrated to enhance immune and antioxidant defenses, increased hepatic HSP70 and SOD activity, elevated total protein, and improved tolerance to acute crowding stress. Furthermore, fish receiving astaxanthin supplementation displayed significantly greater resistance when challenged with *Proteus mirabilis* (Liu et al. 2016).

### Extraction and analysis

Astaxanthin extraction is carried out via solvents such as acetone and ethanol; acids; edible oils such as soybean, maize, olive, and grape seeds; and extraction methods such as microwave-aided, sonication-aided, supercritical fluid and enzymatic techniques (Dong et al. 2014). Additionally, vegetable oils (corn, grape seed, olive, soybean, and corn) were utilized in the extraction from *Hematococcus* cultures, where mixing the biomass with oils enabled astaxanthin to migrate into the lipid phase. Among the tested oils, olive oil achieved the highest recovery rate, reaching 93% (Kang and Sim 2008). 1.3 mg astaxanthin has been successfully extracted from each one gram of *Phaffia rhodozyma* under acidic conditions (Ni et al. 2008). Microwave-assisted extraction at a temperature of 75 °C for 5 min resulted in a yield of 75% astaxanthin that was unfortunately high in the acetone (Storebakken et al. 2004; Ruen-ngam et al. 2010). Supercritical fluid extraction from *Hematococcus* using ethanol and sunflower oil has been known to yield 80–90% astaxanthin (Machmudah et al. 2006; Nobre et al. 2006; Wang et al. 2012a, b). Moreover, astaxanthin was extracted repetitively via solvents, pooled, evaporated via a rotary evaporator, and then redissolved in solvent, after which the absorbance of the extract at 476–480 nm was measured to determine the astaxanthin concentration (Ranga Rao et al. 2010). Furthermore, astaxanthin in the extract can be quantified via HPLC/MS (Ranga et al. 2009).

### Biosynthesis

**Fig. 2:**
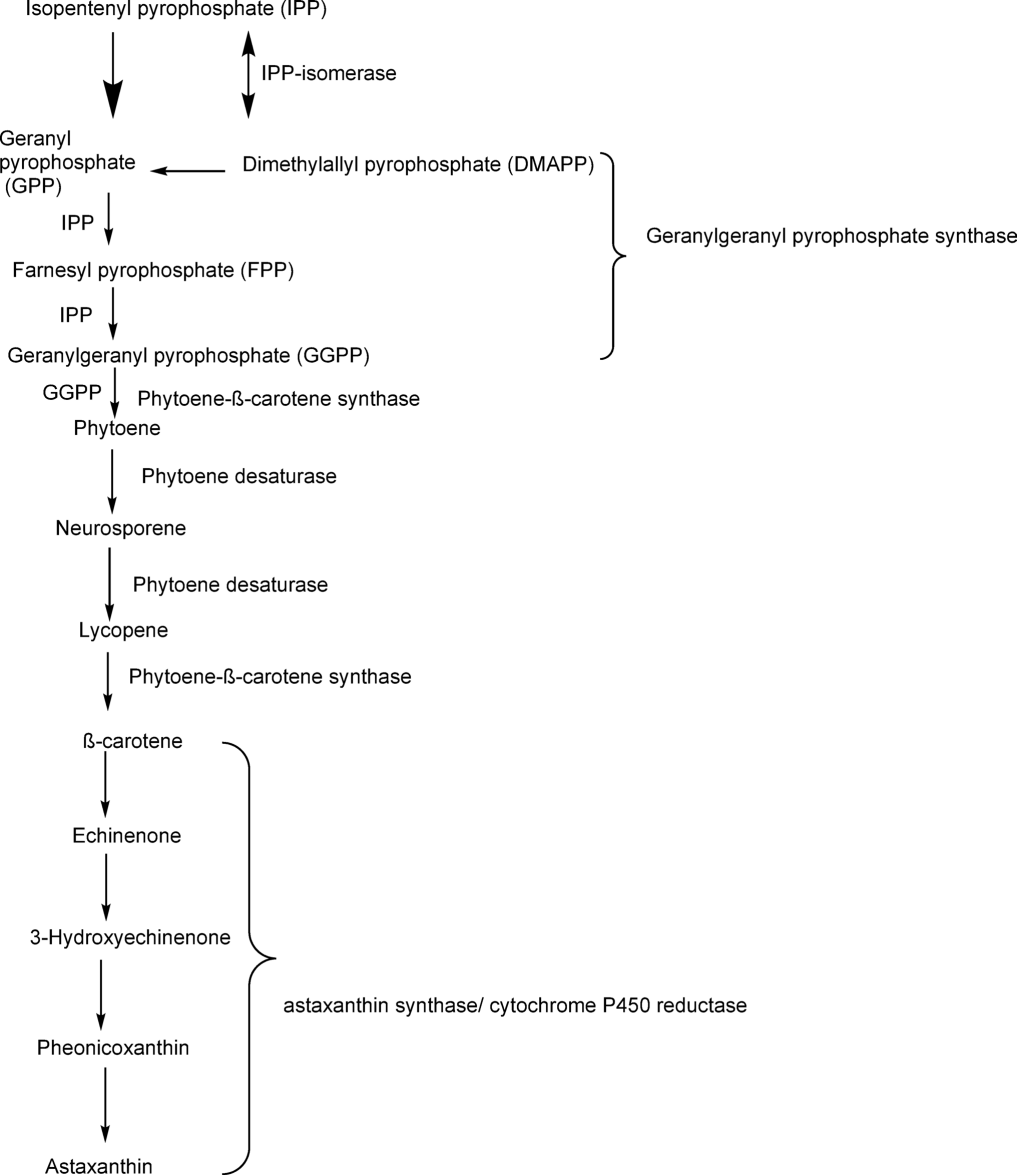
Diagram of the astaxanthin biosynthetic pathway in *Xanthophyllomyces dendrorhous*

Astaxanthin production is a complex process with numerous precursor metabolites. Such process can be divided into 4 phases: (i) precursor metabolite production; (ii) synthesis of isopentenyl diphosphate (IPP) and dimethyl-allyl pyrophosphate (DMAPP); (iii) synthesis of β-carotene from IPP and DMAPP; and (iv) synthesis of astaxanthin from β-carotene (Andrewes et al. 1976; An et al. 1999; Fang et al. 2019) (Fig. 2).

## Pharmacological activities

### Overview of astaxanthin as a potent natural antioxidant and anti-inflammatory agent

Astaxanthin is recognized for its strong antioxidant and anti-inflammatory characteristics (Chang and Xiong 2020; Hwang et al. 2020). Astaxanthin exhibits significant activity due to its distinctive chemical structure, characterized by an extended conjugated double-bond system that enables efficient quenching of reactive oxygen species (ROS) (Nishida et al. 2023; Dembitsky 2024). Furthermore, a unique characteristic of astaxanthin is its ability of integrating itself into the lipid bilayer without compromising the integrity of the plasma membrane. This allows for effective integration into cell membranes, thereby preserving cellular structures from oxidative damage (Nair et al. 2023).

The remarkable antioxidant effects of astaxanthin has drawn much attention lately as they are thought to outperform other common carotenoids, including beta-carotene and lycopene, besides conventional antioxidants (Stepnowski et al. 2004; Miyashita 2014). The increased efficiency of astaxanthin is a result of its capacity to stabilize free radicals by transferring excess electrons along its extended conjugated system, thereby neutralizing reactive molecules—a process that many antioxidants undergo. Additionally, the structure of astaxanthin allows it to cross lipid bilayers, providing comprehensive protection across cell membranes (Chae et al. 2022; Zhang et al. 2025).

Furthermore, astaxanthin stimulates and enhances natural antioxidant defense mechanisms in cells. It has been demonstrated to increase the activity of key antioxidant enzymes. These enzymes play important roles in molecular defense by detoxifying superoxide radicals into less damaging molecules, limiting oxidative damage at the mitochondrial level, where the majority of cellular ROS are produced (Hormozi et al. 2019; Balendra and Singh 2021; Heng et al. 2021; Demirci-Çekiç et al. 2022; Liu et al. 2022; Taheri et al. 2022). By enhancing these antioxidant defenses, astaxanthin preserves mitochondrial health, which is essential for energy generation and general cellular function. The influence on these enzymes is particularly significant in metabolically active organs, where the protective properties of astaxanthin reduce cell death and enhance organ function under oxidative stress conditions (Liu et al. 2009; Nan et al. 2019; Sztretye et al. 2019).

In addition to its antioxidative character, astaxanthin has anti-inflammatory actions by regulating key inflammatory mechanisms and diminishing the production of proinflammatory mediators (Kohandel et al. 2022). Inflammation is a multifaceted cellular response mechanism that involves several signaling molecules and transcription factors (Saha et al. 2020). Astaxanthin can downregulate inflammatory mediators that are fundamental in chronic inflammation and disease progression (Wu et al. 2024). This anti-inflammatory property was validated in various animal models that target the liver, kidneys, central nervous system (CNS), and heart.

#### Pharmacological applications of astaxanthin in various health domains

**Fig. 3:**
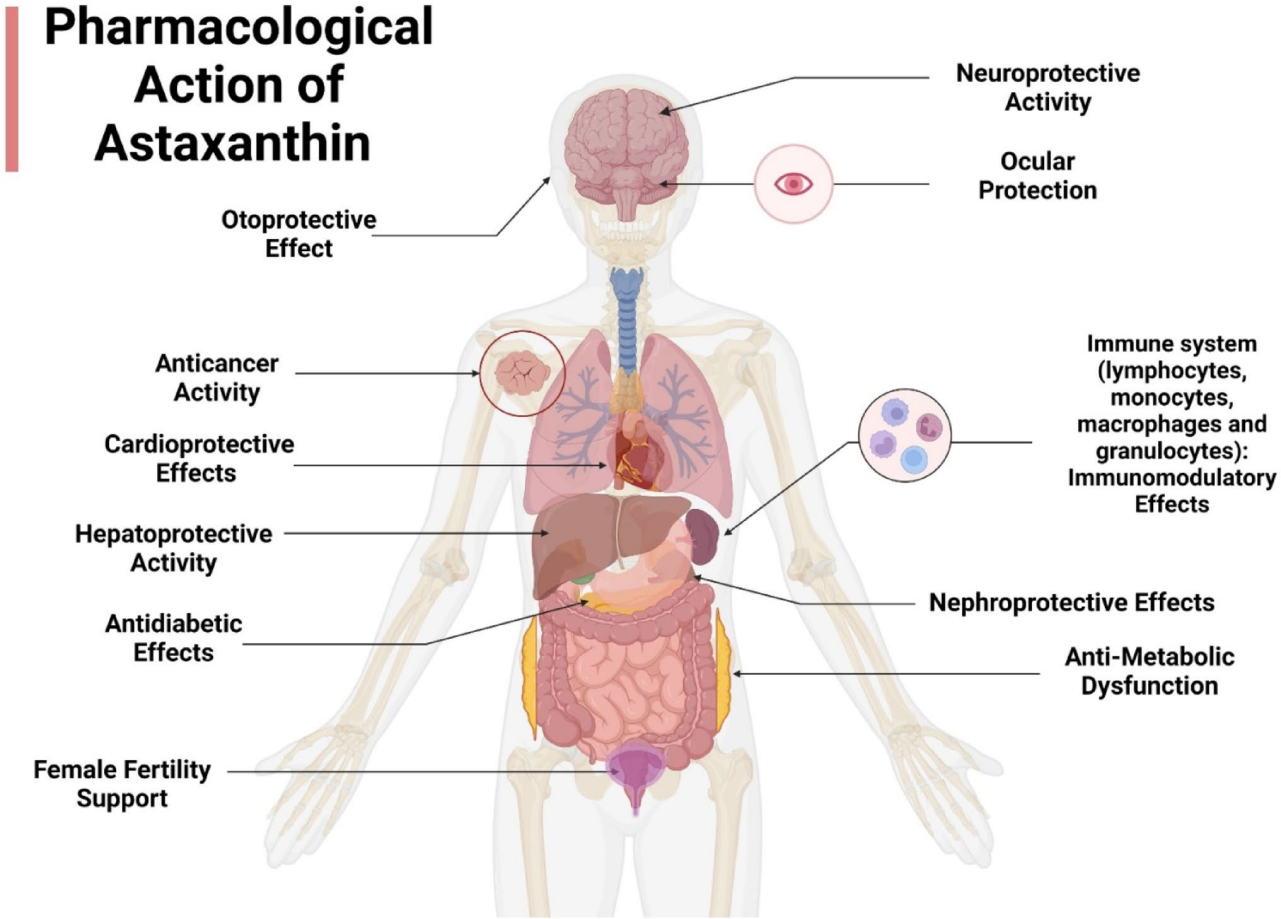
Pharmacological actions of astaxanthin in various health domains. Created in BioRender. Elgindy, A. (2025) https://BioRender.com/0wub0vt

The pharmacological applications of astaxanthin are broad and relevant across various health domains (Fig. 3), primarily owing to its dual capacity to mitigate oxidative stress and inflammation, two fundamental factors in several chronic pathologies. Astaxanthin can also modulate the immune response and mitochondrial functions. Evidence indicates benefits in cardiometabolic regulation, neuroprotection, liver and kidney health, skin photoprotection, immune modulation, and improving reproductive function. Collectively, these multifaceted potentials promote astaxanthin as a promising therapeutic candidate in the complementary interventions for such pathologies. The detailed mechanistic roles of astaxanthin in major physiological systems are discussed in the following sections.

### Cardioprotective effects

**Fig. 4:**
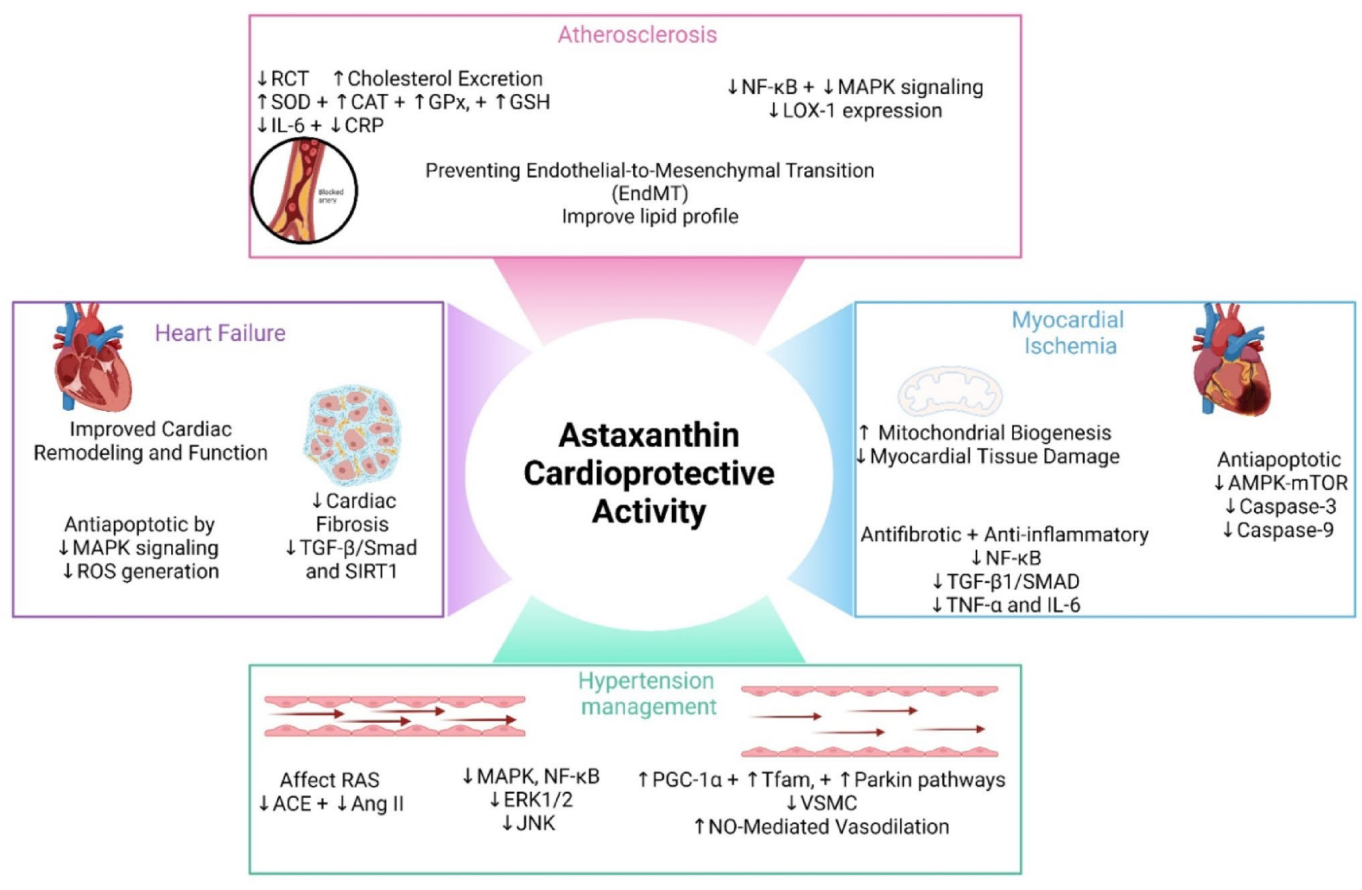
Cardioprotective effects of astaxanthin. Created in BioRender. Elgindy, A. (2025) https://BioRender.com/0wub0vt

Owing to its potent antioxidant and anti-inflammatory properties, astaxanthin has emerged as a promising cardioprotective agent (Fig. 4) (Pashkow et al. 2008). Cardiovascular diseases persist as leading causes of global morbidity and mortality (Ibrahim 2025). This section describes the detailed cardioprotective mechanisms of astaxanthin and corroborates recent evidence from preclinical and clinical studies.

#### Atherosclerosis prevention

Astaxanthin has demonstrated significant cardiovascular protective effects, particularly in atherosclerosis prevention. It enhances reverse cholesterol transport (RCT) by promoting cholesterol efflux from macrophage foam cells through transporters such as ABCA1 and ABCG1, ultimately increasing cholesterol excretion and reducing aortic plaque formation (Zhang et al. 2023a, b). In an animal model, such effect was demonstrated to be mediated by circTPP2/miR-3073b-5p/ABCA1 axis, which regulates cholesterol efflux and foam cell formation, reducing lipid accumulation and preventing atherosclerosis progression (Zou et al. 2017). The prodrug CDX-085 similarly improves lipoprotein profiles and decreases AS in animal models and *in-silico*, suggesting therapeutic potential for cardiovascular diseases (Barizi et al. 2024). These mechanisms underscore the importance of astaxanthin inhibition of foam cell formation and lipid deposition, which are pivotal in atherosclerosis pathogenesis.

In addition to its lipid-lowering properties, astaxanthin provides cardiovascular benefits by lowering oxidative stress as well as inflammation. It enhances antioxidant defenses, improves lipid profiles, and suppresses proinflammatory cytokines (Xu et al. 2014). In pre-clinical studies, the anti-inflammatory actions of astaxanthin were found to involve regulating the NF-κB and MAPK signaling, thereby lowering cytokine levels and increasing nitric oxide (NO) bioavailability. These effects contribute to maintaining endothelial function and preventing endothelial‒mesenchymal transition (EndMT), which is a significant contributor in atherosclerotic plaque progression (Pereira et al. 2021; Barizi et al. 2024). Notably, astaxanthin suppresses the expression of LOX-1, a key receptor that mediates ox-LDL-induced endothelial injury, thereby interrupting the destructive cycle of ROS generation and LOX-1 activation in human umbilical vein endothelial cells (HUVECs) (Zhu et al. 2022).

The multifaceted cardiovascular benefits of astaxanthin also include its impact on gut health and metabolic regulation. Astaxanthin-rich extracts have shown efficacy in reducing body weight, lipid accumulation, and atherosclerotic lesions in mice, while enhancing the composition of the gut microbiota, predominantly in promoting the growth of *Akkermansia uciniphila* (bacteria) and bile acid excretion, which play roles in cholesterol metabolism (Liu et al. 2018a, b). In clinical trials, astaxanthin supplementation was reported to decrease total and LDL-cholesterol in CAD patients, suggesting its potential as a functional supplement or adjunct therapy (Heidari et al. 2023). These characteristics of astaxanthin, along with its favorable safety profile, present it a prospective candidate for preventive and/or adjuvant therapy for cardiovascular disorders (Pereira et al. 2021).

#### Myocardial ischemia

Myocardial ischemia, characterized by inadequate blood supply to the cardiac muscle, results in significant outcomes, including myocardial infarction and ischemia‒reperfusion damage. This condition involves complex pathogenic pathways which impair cardiac function and structure. Oxidative damage, especially in mitochondria, is pivotal in cardiac injury, underscoring the therapeutic importance of therapies aimed at this pathway. Astaxanthin was successful in mitigating oxidative stress, promoting mitochondrial biogenesis, and improving cardiac function while reducing myocardial tissue damage (Zuluaga et al. 2017; Alam et al. 2018; Mahmoud et al. 2024; Shen et al. 2024).

In addition to having antioxidant capabilities, astaxanthin regulates essential molecular processes, including the NF-κB, TGF-β1/SMAD, and AMPK-mTOR signaling cascades. These pathways are pivotal to inflammation, fibrosis, and apoptosis, all of which aggravate myocardial ischemia. Astaxanthin was demonstrated to reduce the concentrations of inflammatory mediators, alleviate myocardial fibrosis by halting the production of collagens I and III, and enhancing cardiac remodeling in experimental models of myocardial infarction (MI) (Shi et al. 2018; Pan et al. 2020; Mahmoud et al. 2024) and in animals models ischemia–reperfusion injury (Hu et al. 2021; Lu et al. 2024). Additionally, it protects myocardial cells in experimental animal models from hypoxic stress by reducing the expression of apoptotic markers such as caspase-3 and caspase-9 and upregulating the expression of beneficial microRNAs including miR-138, further strengthening its cardioprotective potential (Gai et al. 2020; Durukan et al. 2022; Zhang et al. 2023a, b).

Recent advancements encompass mitochondrial-targeted delivery strategies for astaxanthin, including liposomal formulations, which guarantee accurate targeting and extended antioxidant efficacy in compromised cardiac tissues (Gao et al. 2022). These advancements, corroborated by both in vivo and in vitro experimental models, validate the capacity of astaxanthin to suppress ferroptosis, protect mitochondria, and modulate macrophage polarization under ischemic conditions, thereby mitigating cardiac damage and enhancing heart function (Gao et al. 2022; Lu et al. 2024; Shen et al. 2024). This multifaceted approach underscores the promise of astaxanthin as an effective therapeutic agent for addressing myocardial ischemia and related complications.

#### Hypertension management

Astaxanthin has shown considerable therapeutic efficacy in both animal models and clinical trials focused on cardiovascular health. In hypertensive animal models, astaxanthin significantly decreases blood pressure by alleviating oxidative stress and restoring vascular function. In spontaneous hypertensive rats (SHRs), astaxanthin enhances mitochondrial function by decreasing ROS generation, augmenting mitophagy, and facilitating mitochondrial biosynthesis through the activation of the PGC-1α, Tfam, and parkin signaling. It also hinders mitochondrial fission by decreasing Drp1 phosphorylation, thus inhibiting vascular smooth muscle cell (VSMC) proliferation and minimizing aortic wall fibrosis (Hussein et al. 2005a, b; Chen et al. 2020). Furthermore, astaxanthin enhances NO-mediated vasodilation and inhibits angiotensin-II (Ang II)-induced vasoconstriction, demonstrating its dual impact on vascular tone and reactivity (Hussein et al. 2005a, b; Preuss et al. 2009).

Further mechanistic insights indicate that astaxanthin regulates systemic inflammation and oxidative stress by modulating critical pathways, including the MAPK, NF-κB, and renin‒angiotensin system (RAS) pathways. Astaxanthin attenuated inflammation and ROS production in the hypothalamic paraventricular nucleus (PVN) of salt-sensitive prehypertensive animals by reducing NOX2/NOX4 expression and phosphorylated ERK1/2 and JNK levels. It further elevates the expression of antioxidant catalase and superoxide dismutase, as well as anti-inflammatory IL-10, thus effectively reversing neuroinflammation induced by hypertension (Gao et al. 2021a, b). Similarly, astaxanthin suppressed RAS activity in Zucker Fatty Rats, decreased angiotensin-converting enzyme (ACE) levels, enhanced vascular function, and reduced the circulating levels inflammatory mediators, including TNF-α and MCP-1 (Preuss et al. 2009, 2011).

Clinical trials have further validated the therapeutic effectiveness of astaxanthin. For patients with type 2 diabetes, 8 weeks of astaxanthin supplementation improved glycemic management, lowered systolic blood pressure, and improved lipid profiles by increasing HDL cholesterol and decreasing triglyceride and very-low-density lipoprotein levels. This was accompanied by a decrease in the levels of oxidative stress markers such as MDA and an increase in antioxidant capacity, highlighting the role of astaxanthin in combating metabolic and cardiovascular risks (Mashhadi et al. 2018; Jabarpour et al. 2024a, b).

#### Heart failure protection

Owing to its antioxidant, mitochondrial enhancing, and anti-inflammatory characteristics, astaxanthin was reported to improve heart failure in both preclinical and clinical reports. Astaxanthin modulates the NF-κB, ROCK II, and caspase pathways, inhibiting apoptosis and decreasing the pro-inflammatory mediators. This improved cardiac remodeling and function in experimental animals (Nakao et al. 2010; Xuan et al. 2016; AlQahtani et al. 2019). Astaxanthin reduces oxidative stress, a significant component in cardiac remodeling and heart failure development, by reducing ROS generation and increasing the levels of antioxidant enzymes. Reports in rats with induced cardiotoxicity have shown that astaxanthin preserves mitochondrial integrity by preventing permeability transition pore (mPTP) opening and improving respiratory chain complex activities, reducing oxidative damage and enhancing ATP synthesis (Baburina et al. 2019; Krestinin et al. 2020; Krestinina et al. 2020). Astaxanthin reduces fibrosis in experimental animals by altering essential fibrotic pathways, such as the TGF-β/Smad and SIRT1 signaling axes, besides lowering the pro-inflammatory cellular mediators (Zhang et al. 2017a, b; Shatoor and Al Humayed 2021; Sarker et al. 2024).

Experimental models have reported cardioprotective effects on conditions such as diabetic cardiomyopathy, hypertensive cardiac remodeling, and doxorubicin-induced cardiotoxicity for astaxanthin (Nakao et al. 2010; Xuan et al. 2016; AlQahtani et al. 2019). Astaxanthin increases heart function by decreasing inflammatory and oxidative stress indicators, lipid peroxidation, and myocardial fibrosis. These effects are associated with the inhibition of MAPK signaling as well as the suppression of ER stress-mediated apoptosis (Zhang and Xu 2018a, b; Wang et al. 2021; Sarker et al. 2024). In isoproterenol-induced cardiac injury models, astaxanthin improved mitochondrial function, restored antioxidant enzyme activities, and protected against Ca^2+^-induced mitochondrial damage, indicating its potential as a mitochondrial-targeted therapeutic agent (Krestinin et al. 2020; Krestinina et al. 2020, 2021).

Clinical trials have shown that astaxanthin can enhance cardiac functionality and consequently the quality of life in heart failure patients. Pilot studies revealed that astaxanthin supplementation improved LVEF and exercise tolerance in individuals with left ventricular systolic dysfunction while also lowering the oxidative stress biomarkers. The antioxidant and anti-inflammatory activities of astaxanthin was linked to improved health-related quality of life (Kato et al. 2020a, b; Ishiwata et al. 2021). Astaxanthin supplementation in randomized controlled trials (RCTs) has been shown to significantly reduce inflammatory marker and lipid profiles, improve endothelial function, and alleviate symptoms of heart failure, highlighting its therapeutic potential (Mohammadi et al. 2024).

The ability of astaxanthin to improve mitochondrial efficiency is crucial to its therapeutic benefits. It improves the mitochondrial membrane potential, Ca^2+^ retention capacity, and mitochondrial protein expression, which are essential for maintaining cardiac function during stress. The protective effects of astaxanthin against oxidative stress extend to brain mitochondria in rats after heart failure, highlighting its systemic advantages in reducing ischemia and oxidative damage associated with cardiovascular conditions (Krestinina et al. 2021; Krestinin et al. 2022; Baburina et al. 2023).

The cardioprotective potential of astaxanthin was strongly supported by both experimental and clinical evidence, primarily through its antioxidant and anti-inflammatory effects that managed to preserve endothelial integrity and modulate lipid metabolic cascades. However, most findings are derived from preclinical models with variable dosing regimens, making direct extrapolation to human cardiovascular disease uncertain. Further clinical validation with standardized formulations and long-term outcomes is warranted.

### Immunomodulatory effects

Astaxanthin has immunomodulatory effects via many molecular mechanisms, including decreasing oxidative stress, regulating cytokines, and modulating immune cells. Its antioxidant capabilities arise from its capacity to neutralize ROS and stabilize cellular membranes, thereby safeguarding immune cells from damage inflicted by increased oxidative stress (Park et al. 2010; Yamashita 2015; Lee 2023; Maiuolo et al. 2023). The antioxidant effects support the immunomodulatory function of astaxanthin, especially in enhancing the synthesis of essential cytokines, including interferon-gamma (IFN-γ) and IL-2. Such cytokines are critical for activating lymphocytes and natural killer cells, hence augmenting the immune system’s capacity to fight infections and tumors (Lin et al. 2016). Furthermore, astaxanthin directly affects the expression of NF-κB, a transcription factor pivotal to inflammatory and immunological responses, thereby diminishing proinflammatory cytokine production and promoting an anti-inflammatory environment (Lee et al. 2003a, b; Park et al. 2010).

The capacity of astaxanthin to modulate neuroinflammatory responses has been studied. It is proposed to modulate immunological pathways influenced by neurotoxic drugs, as demonstrated in a study assessing its effects on neuroinflammation induced in mice by potassium channel blockers. This regulatory impact highlights its extensive potential in managing conditions related to immunological dysregulation (Sifi et al. 2016). Moreover, the integration of astaxanthin into cellular systems, particularly immune cells, has been demonstrated to augment immunological function, as evidenced by its capacity to increase immunoglobulin production in fish models (Eldessouki et al. 2024).

The ability of astaxanthin to mitigate cytokine storms, such as those observed in severe COVID-19 cases, involves the downregulation of cytokine overexpression and the attenuation of oxidative stress-induced damage. It accomplishes this by inhibiting the Janus kinase-signal transducer and activator of transcription (JAK-STAT) pathway, which is involved in cytokine signaling. Additionally, astaxanthin indirectly modulates cytokine activity by reducing ROS production at its source and influencing mitochondrial function in immune cells. Astaxanthin has been demonstrated to prevent systemic inflammation by moderating the balance between pro-inflammatory and anti-inflammatory cytokines, including IL-10, and by reducing oxidative stress markers (Talukdar et al. 2020).

The notable immunomodulatory properties exerted by astaxanthin are primarily mediated via attenuating pro-inflammatory cytokines and enhancing antioxidant responses. Despite consistent in vitro and pre-clinical data, human studies often lack mechanistic clarity and standardized outcome measures. Future investigations should integrate immune profiling and translational endpoints to delineate its clinical immunomodulatory potential.

### Hepatoprotective activity

**Fig. 5:**
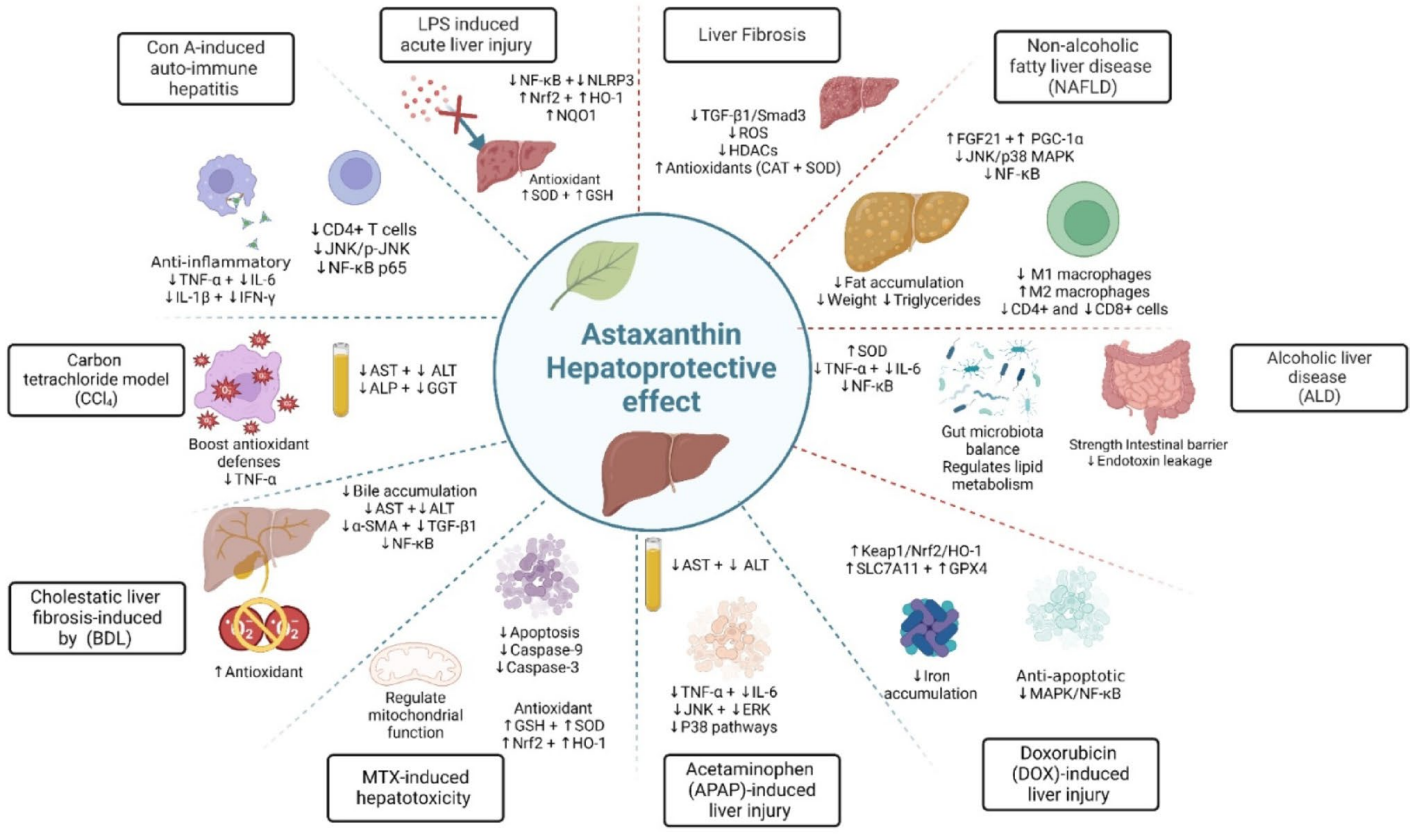
Hepatoprotective activity of astaxanthin. Created in BioRender. Elgindy, A. (2025) https://BioRender.com/0wub0vt

Astaxanthin possesses several significant properties, including free radical neutralization, and the regulation of numerous signaling pathways that may contribute to the prevention and treatment of liver-related pathologies such as liver injury, fibrosis, and cancer (Fig. 5) (Arefpour et al. 2024).

#### Liver fibrosis

In human hepatic stellate cells (HSCs), LX-2, astaxanthin decreases ROS, and activates antioxidant enzymes. Astaxanthin blocks fibrosis-related pathways, such as the TGF-β1/Smad3 pathway, and induces apoptosis in fibrotic cells, helping to prevent excess extracellular matrix formation. Additionally, it regulates histone deacetylases (HDACs), which contribute significantly in fibrosis, and encapsulation techniques improve its stability for therapeutic use (Shen et al. 2025).

#### Nonalcoholic fatty liver disease (NAFLD)

NAFLD is a chronic hepatic pathology that is characterized by excessive accumulation of fat in the functional hepatocytes irrespective of alcohol consumption or other liver toxins. It primarily results from disrupted hepatic metabolism of free fatty acids (Wu et al. 2020; Powell et al. 2021). In 2007, Ikeuchi and colleagues examined how astaxanthin impacted obese animals that were fed on high-fat diet. They reported that astaxanthin prevented increases in body weight and fat accumulation due to the diet while also lowering liver weight, liver triglycerides, plasma triglycerides, and total cholesterol (Ikeuchi et al. 2007). Moreover, a previous in vivo report about high-fat diet fed mice showed that astaxanthin can upregulate fibroblast growth factor 21 (FGF21) and peroxisome proliferator-activated receptor-γ coactivator-1α (PGC-1α), which are critical for improving lipid metabolism and mitochondrial biogenesis (Wu et al. 2020).

Inflammation also contributes primarily to NAFLD development. Research by Ni et al. revealed that astaxanthin significantly reduces the occurrence of pro-inflammatory M1 macrophages while increasing the anti-inflammatory M2 subtype in the liver of experimental animals. It also decreased liver infiltration by CD4 + and CD8 + cells, effectively reducing inflammation., astaxanthin was found to be more effective at lowering lipid accumulation, enhancing insulin signaling, and inhibiting proinflammatory pathways, particularly the JNK/p38 MAPK and NF-κB pathways, when compared to vitamin E (Ni et al. 2015). Similarly, astaxanthin was found to reduce macrophage infiltration in the liver and fat tissue, decreased inflammation and fibrosis, and improved mitochondrial fatty acid oxidation in the muscle tissue of obese mice (Kim et al. 2017). Such results propose that astaxanthin may benefit lipid metabolism, providing a foundation for further research into NAFLD treatment.

#### Alcoholic liver disease (ALD)

ALD results from prolonged excessive alcohol ingestion, leading to a variety of liver injuries ranging from steatosis to hepatitis, fibrosis, cirrhosis, and finally hepatocellular carcinoma (HCC). Alcohol metabolism generates acetaldehyde and ROS, leading to increased oxidative stress, lipid peroxidation, and mitochondrial dysfunction. These processes disrupt liver cell structure and function, activate inflammatory pathways (e.g., NF-κB), and promote apoptosis and necrosis. Additionally, alcohol alters the gut microbiota, increasing intestinal permeability and allowing endotoxins to enter the bloodstream, exacerbating liver inflammation and injury. Astaxanthin protects against alcohol-induced liver damage in mice through several mechanisms. It acts as a potent antioxidant, reducing ROS and enhancing the levels of antioxidant enzymes. Astaxanthin has anti-inflammatory effects, suppresses cytokines and inhibits NF-κB activation. Moreover, it improves the gut microbiota balance, strengthens the intestinal barrier and reduces endotoxin leakage. Additionally, astaxanthin regulates lipid metabolism, preventing liver fat accumulation. It also protects liver histology by reducing steatosis, necrosis, and inflammation (Liu et al. 2018a, b).

#### Drug-induced liver injury

##### Lipopolysaccharide (LPS)-induced acute liver injury

LPS, a bacterial endotoxin, is a key mediator of liver disease and contributes to acute liver injury through excessive immune activation and inflammatory cytokine release. LPS, a bacterial endotoxin, is a key mediator of liver disease and contributes to acute liver injury (ALI) through excessive immune activation and inflammatory cytokine release. LPS triggers oxidative stress, inflammation, and hepatocyte apoptosis through pathways such as NF-κB, NOD-like receptor family pyrin domain containing 3 (NLRP3) inflammasome activation, and excessive production of ROS. These mechanisms lead to elevated levels of proinflammatory cytokines, along with mitochondrial dysfunction, lipid peroxidation, and hepatocyte necrosis. LPS-induced damage disrupts liver function, as evidenced by increased liver enzymes and bilirubin, and promotes the progression of liver diseases such as fibrosis and cirrhosis​ (He et al. 2023).

Astaxanthin as a potent antioxidant counters LPS-induced liver injury in experimental animals by regulating key molecular pathways. Astaxanthin inhibits the NF-κB and NLRP3 signaling cascades, decreasing inflammation by lowering proinflammatory cytokines and minimizing inflammatory cell infiltration in the liver. It enhances the antioxidant defense system by activating Nrf2 and increasing the expression of downstream protective enzymes such as HO-1 and NQO1. Astaxanthin also alleviates oxidative stress by scavenging ROS and restoring the activity of antioxidant enzymes. Moreover, astaxanthin reduces hepatocyte apoptosis by preserving mitochondrial integrity and suppressing lipid peroxidation. These actions help restore liver structure and function, offering protection against LPS-induced liver damage (He et al. 2023). Moreover, a previous study demonstrated that the encapsulation of astaxanthin within liposomes effectively mitigated LPS-induced liver toxicity in experimental animals by lowering the levels of inflammation markers in rats (Chiu et al. 2016).

##### Concanavalin A (Con A)-induced autoimmune hepatitis (AIH)

Con A is a plant-derived lectin known to stimulate cell division. It strongly activates CD4 + T cells and macrophages within the hepatic sinus, triggering their proliferation and promoting the release of pro-inflammatory mediators (Ibrahim et al. 2023). Previous evidence in high fat-fed mice has shown that astaxanthin is capable of terminating the inflammatory response (Bhuvaneswari et al. 2014). Similarly, in a RCT, astaxanthin exerted its anti-inflammatory effect via reducing the release of TNF-α, IL-6, IL-1β and IFN-γ (Fassett et al. 2008a, b). Con A can initiate the nuclear translocation and subsequent activation of NF-κB which will then increase the downstream pro-inflammatory mediators (Ibrahim et al. 2023). In LPS-stimulated RAW264.7 cells and primary macrophages as well as in LPS-administrated mice, astaxanthin abolished the NF-κB activation triggered by Con A, thereby lowering its nucleus expression and reducing inflammation (Lee et al. 2003a, b). Earlier reports showed that the pathogenesis of liver injury induced by Con A involves apoptosis and autophagy. Fortunately, astaxanthin also reduces apoptosis and autophagy regulated by the JNK/p-JNK pathway, playing a protective role in Con A-induced autoimmune hepatitis in BALB/C mice (Li et al. 2015).

##### Carbon tetrachloride model (CCl_4_)

A previous study demonstrated the hepatoprotective activity of astaxanthin in counteracting liver damage caused by CCl_4_, a toxic compound. Astaxanthin, a natural antioxidant, helps reduce oxidative stress and inflammation in an in vivo model of liver damage induced by CCl_4_. By neutralizing harmful free radicals and increasing antioxidant defenses, astaxanthin can prevent cellular damage, support liver function, and potentially aid in the recovery process from liver injury induced CCl_4_ (Rao et al. 2015; Islam et al. 2017). Moreover, a previous study reported that astaxanthin was protective against hepatic damage in rats with hepatocellular carcinoma (HCC) induced by CCl_4_. Treatment with astaxanthin significantly reduced the elevated levels of liver enzymes, bilirubin, and the inflammatory marker TNF-α, all of which were high in untreated HCC rats. This reduction indicates that astaxanthin helps restore normal liver function and reduce inflammation, supporting its potential as a liver-protective remedy in HCC (Johnson and Modo 2024).

##### Cholestatic liver fibrosis induced by bile duct ligation (BDL)

Bile duct ligation (BDL) induces oxidative stress, liver injury, and inflammation, as shown by decreased activities of antioxidant enzymes and elevated levels of liver enzymes, lipids, and fibrosis markers (α-SMA and TGF-β1). Astaxanthin, with its strong antioxidant properties, increases antioxidant enzyme activity, reduces liver enzyme and lipid levels, decreases fibrosis markers, and mitigates histopathological damage. These findings suggest that astaxanthin protects liver structure and function, counteracting oxidative damage, inflammation, and fibrosis in cholestatic injury in adult wistar rats (Laderian et al. 2024). In an in vivo model of cholestatic liver fibrosis induced by BDL, bile accumulation led to excessive oxidative stress and inflammatory responses, promoting HSC activation and extracellular matrix deposition. Astaxanthin attenuated these pathological changes by restoring antioxidant enzyme activity, reducing the expression of α-SMA and TGF-β1, while modulating the NF-κB and TGF-β1 signaling pathways, thereby mitigating fibrosis progression in cholestatic liver injury in mice (Shen et al. 2014).

##### Methotrexate (MTX)-induced hepatotoxicity

MTX is an effective treatment for cancer and autoimmune diseases, but its use is significantly limited by its hepatotoxicity. MTX induces liver damage in experimental *vivo* models through oxidative stress by weakening antioxidant defenses through depleting GSH and increasing MDA. In addition, MTX causes mitochondrial dysfunction and enhances hepatocyte apoptosis. It activates apoptotic markers such as caspase-9 and caspase-3 and disrupts the balance between the pro-apoptotic Bax and anti-apoptotic Bcl-2. These processes lead to liver cell death, elevated levels of liver enzymes in the bloodstream, and reduced expression of protective genes such as Nrf2 and HO-1. However, treatment with astaxanthin increased the expression of protective genes such as Nrf2 and HO-1. Furthermore, it mitigates apoptosis by downregulating caspase-9 and caspase-3, regulates mitochondrial function by reducing cytochrome c release, and preserves the membrane potential. In addition, astaxanthin enhances antioxidant defenses by restoring enzymes such as GSH and SOD. These combined effects improve liver histopathology, reduce inflammation, and protect liver integrity from MTX-induced damage (Hamdan et al. 2022a, b; Azadian et al. 2024).

##### Paracetamol (Acetaminophen)-induced liver injury

Astaxanthin has protective effects against paracetamol (acetaminophen)-induced liver injury by reducing the levels of liver damage markers, improving antioxidant defenses, and lowering oxidative stress in experimental animals. Astaxanthin decreases the levels of ROS, lipid peroxidation, and proinflammatory cytokines. It also reduces hepatocyte necrosis and inflammation by inhibiting the JNK, ERK, and P38 pathways, which are involved in cell death and inflammation. Such actions underscores the ability of astaxanthin to protect the liver from paracetamol (acetaminophen) toxicity through its strong antioxidant and anti-inflammatory effects (Zhang et al. 2017a, b).

##### Doxorubicin (DOX)-induced liver injury

Hepatotoxicity is a notable side effect during patient therapy with DOX due to its accumulation in the liver (Yin et al. 2024). According to a previous study, more than 30% of breast cancer patients experience hepatic injuries after receiving DOX (Damodar et al. 2014). It causes liver injury through mechanisms including oxidative stress, inflammation, and ferroptosis, characterized by iron accumulation, lipid peroxidation, and mitochondrial dysfunction; moreover, research has indicated that DOX disrupts the nuclear entry of topoisomerase II, hindering DNA replication (Tewey et al. 1984). These processes lead to hepatocyte damage, elevated liver enzyme levels, and the release of inflammatory cytokines (Yin et al. 2024).

Numerous preclinical reports emphasized the hepato-protective actions of astaxanthin (Li et al. 2020a, b). Astaxanthin combats DOX-induced hepatotoxicity by alleviating oxidative stress, inflammation, and ferroptosis. It reduces ROS, activates the antioxidant enzymes, and prevents lipid peroxidation. Additionally, astaxanthin induces the Keap1/Nrf2/HO-1 pathway, a key signaling mechanism in cellular antioxidant defense. By promoting Nrf2 nuclear translocation and upregulating the downstream antioxidant proteins, astaxanthin effectively mitigates oxidative damage in mice (Ma et al. 2020).

Moreover, astaxanthin minimizes iron accumulation, a key factor in ferroptosis, by regulating iron metabolism proteins such as ferritin and transferrin receptors. It also influences critical ferroptosis-related pathways, including SLC7A11 and GPX4, safeguarding liver cells from damage caused by DOX. Additionally, astaxanthin mitigates inflammation by reducing the levels of pro-inflammatory cytokines such as TNF-α and IL-1β, suppressing MAPK and NF-κB pathway activation, and decreasing hepatocyte apoptosis. Additionally, it improves the liver’s histopathology and restores liver integrity in experimental models. Owing to its collective antioxidant, anti-inflammatory, and anti-apoptotic characters, astaxanthin is a promising hepatoprotective agent that reduces DOX-related liver damage while preserving its anticancer effectiveness (Ma et al. 2020; Alkabbani et al. 2024; Yin et al. 2024).

The studies reviewed in this section consistently demonstrate that astaxanthin ameliorates hepatic oxidative stress, steatosis, and fibrosis via modulation of several hepatoprotective signaling pathways including Nrf2, NF-κB, and PPAR cascades. Yet, much of the evidence stems from animal models without any clinical confirmations. Human trials using well-defined endpoints and quantifiable biomarkers are mandatory to establish translational relevance.

### Neuroprotective activity

Since astaxanthin can pass the BBB (Zhang et al. 2015), the brain has been considered the primary target organ where astaxanthin can ameliorate acute neuronal injuries and chronic neurodegeneration (Wu et al. 2015; Galasso et al. 2018). Recently, the biomedical benefits of astaxanthin have earned significant attention from neuroscientists. Evidence suggests that astaxanthin is effective against neurodegenerative pathologies as well as ischemic injuries (Bahbah et al. 2021).

#### Alzheimer’s disease (AD)

The most debilitating neurological illness affecting elderly individuals is AD. Although the precise root etiology of AD is unknown, the pathologic features of AD include age-related dementia and inflammatory injuries, which are characterized by abnormal intracellular buildup of hyperphosphorylated tau proteins creating neurofibrillary tangles (NFTs) inside neurons and abnormal extracellular accumulation of misfolded amyloid-β (Aβ) proteins (Karimi Askarani et al. 2020; Oliyaei et al. 2023) as a result of homeostatic imbalance between the synthetic process and the clearance mechanisms (Wildsmith et al. 2013). In addition to plaque and tangle buildup and mitochondrial dysfunction, cerebral amyloid angiopathy (CAA), synaptic failure, oxidative damage, and neuroinflammation are key contributors to the pathophysiology of this disease. As a result, antioxidant therapy could serve as a therapeutic and preventive approach to bolster the body’s natural antioxidant defenses.

Literature indicates that astaxanthin may mitigate AD symptoms by inhibiting enzymes such as AChE, BuChE, BACE-1, and MAO, which are involved in disease pathology. Additionally, it helps reduce Aβ accumulation, a hallmark of AD, while preventing the apoptosis triggered by Aβ and oxidative stress-induced cytotoxicity by modulating antioxidant enzymes and suppressing the ERK pathway (Oliyaei et al. 2023). Babalola et al. (2023) reported that astaxanthin enhanced Aβ clearance, stimulated the production of anti-inflammatory proteins, and reduced cytokine levels by activating autophagy in porcine brain capillary endothelial cell (pBCEC) cultures. Moreover, previous studies have shown that cognitive impairments in an AD rat model were improved following treatment with astaxanthin. Supplementing with docosahexaenoic acid-acylated astaxanthin diesters (AST-DHA) helped to ameliorate cognitive dysfunction in a double-transgenic mouse model (Kohandel et al. 2022). Moreover, a previous study has explored the neuroprotective properties of astaxanthin against glutamate-induced toxicity in mouse hippocampal HT22 cells, highlighting its action via Nrf2-dependent HO-1 expression (Wen et al. 2015). Astaxanthin supports cellular defense mechanisms, making it a promising natural compound for AD treatment (Oliyaei et al. 2023).

#### Parkinson’s disease (PD)

PD is an age-related common neurodegenerative disorder, with key neuropathological features including the presence of Lewy bodies containing α-synuclein (SNCA), the degeneration of dopaminergic neurons, and damage to non-dopaminergic neurons in the substantia nigra (Zhang et al. 2015). The SNCA buildup is closely linked to disease progression and contributes to both motor and nonmotor symptoms (Bahbah et al. 2021). Nearly all genes implicated in PD are regulated by miRNAs (Leggio et al. 2017). In substantia nigra, a decrease in miR-7 is linked with SNCA buildup and the loss of dopaminergic neurons (Bahbah et al. 2021). Astaxanthin can infer neuroprotection against PD through multiple mechanisms, including reducing dopaminergic neuron degeneration. These effects are mediated by the miR-7/SNCA pathway, which subsequently inhibits endoplasmic reticulum (ER) stress (Shen et al. 2021). In an in vitro study, Shen et al. (2021) reported that astaxanthin mitigated MMP-induced apoptosis and ER stress while also correcting the dysregulation of miR-7 and SNCA expression in SH-SY5Y cells. Moreover, the anti-inflammatory effects of astaxanthin contribute to preventing CNS pathologies progression. Astaxanthin inhibits the NF-κB-dependent signaling cascades; it also reduces the downstream inflammatory mediators. In addition, astaxanthin increases antioxidant enzyme activities and reduces ROS in different regions of the CNS of experimental animals (Al-Amin et al. 2015).

#### Depression

Depression is a prevalent and complex psychological disorder that affects human health (Zhou et al. 2017). Recent evidence suggests a strong link between depression and oxidative stress/inflammation, with non-monoaminergic pathways playing a significant role, making them a focus of current research. Additionally, the damaging intracellular mechanisms of oxidative stress and inflammation are key factors contributing to depression and anxiety (Ke et al. 2020).

Numerous reports highlighted the antidepressant properties of astaxanthin in various animal models. Zhou and colleagues showed that astaxanthin mitigated hyperglycemia-induced neuroinflammation, a factor contributing to depression. They further demonstrated that astaxanthin exerted antidepressant-like effects in diabetic mice by reducing the levels of IL-1β, IL-6, cyclooxygenase-2 (COX-2), cleaved caspase-3, and GFAP while preserving neuronal structures in the hypothalamus, hippocampus, and amygdala of mice (Zhou et al. 2017). Chronic term treatment with trans-astaxanthin (trans-AST) effectively prevents comorbid depression in mice because of its potent anti-inflammatory effect and its role in modulating the serotonergic pathway (Jiang et al. 2018); thus, it may be a strong antidepressant drug.

#### Multiple sclerosis (MS)

MS is an autoimmune disorder exhibiting a chronic inflammatory course that frequently impacts young adults and is nontraumatic in nature (Ghaiad et al. 2020). The main cause of demyelination, a chief contributor to the pathogenesis of MS, is the death of oligodendrocytes triggered by focal immune cell activation. As a result, antioxidants could serve as co-adjuvant treatments to protect against oligodendrocyte death and mitigate complications related to demyelination (Lotfi et al. 2021). Lotfi et al. (2021) demonstrated that administering astaxanthin reduced oligodendrocyte loss and myelin sheath damage while also increasing muscle strength, as evidenced by improved performance in the basket behavior test in a cuprizone model of MS.

### Anticancer activity

The biological activity of astaxanthin within the body provides numerous benefits, including its anticancer properties (Erzurumlu et al. 2023). Earlier reports have examined the impact of astaxanthin on different types of cancer in mammals (Kim et al. 2015; Zhang and Wang 2015). Research indicates that astaxanthin can hinder cancer progression by inducing apoptosis, restricting uncontrolled cell growth, and suppressing tumor cell migration and invasion (Shanmugapriya et al. 2019; Erzurumlu et al. 2023).

Previous studies have examined the impact of astaxanthin both in vivo and in vitro on different types of cancer in mammals (Kim et al. 2015; Zhang and Wang 2015). Various preclinical reports showed that astaxanthin can inhibit the development of liver carcinoma (Bal et al. 2023), oral cancer (Tanaka et al. 1995), colon cancer (Nagendraprabhu and Sudhandiran 2011), and breast cancer (Zeini et al. 2024) and may help suppress the growth of prostate cancer (PCa) aggressive DU145 cells (Sun et al. 2020). Another in vitro research indicated that the antitumor potential of astaxanthin nanoemulsions exerted via inducing apoptosis, restricting uncontrolled cell growth, and suppressing tumor cell migration and invasion (Shanmugapriya et al. 2019).

Astaxanthin can provide neuroprotection by mitigating oxidative stress, modulating mitochondrial function, and suppressing neuroinflammation. These effects have been observed in vitro and in animal models of several neurodegenerative diseases. Nonetheless, human data remain preliminary, and variability in dosing and delivery forms challenges consistency. More robust clinical trials are needed to validate these neuroprotective effects.

#### Direct effects of astaxanthin on cancer

##### Induction of apoptosis

Apoptosis, a pre-planned cell death mechanism, is indispensable for development, maintaining homeostasis, and defending against cellular damage and mutations. Though, disproportionate apoptosis can disrupt balance and contribute to various pathologies. Such mechanism is controlled by the Bcl-2 family, which includes pro-apoptotic factors and anti-apoptotic factors. Following apoptotic signals, Bad and Bax facilitate mitochondrial cytochrome c release, joining protease activator-1 and caspase-9 that activates caspase-3, initiating apoptosis (Hormozi et al. 2019).

Under normal conditions, carotenoids can ameliorate cellular oxidative stress. However, in cancers with distinctively increased intracellular ROS contents, carotenoids can be pro-oxidants and initiate a ROS-mediated apoptotic mechanism in cancer cells (Shanmugapriya et al. 2019; Erzurumlu et al. 2023). Moreover, a previous study revealed that astaxanthin may induces apoptosis via mitochondrial pathway in a human ovarian cancer cell line (SKOV3) by upregulating Bcl-2/Bax expression and subsequently caspase-3 activation (Si and Zhu 2022).

##### Inhibition of uncontrolled cell proliferation

P53, often called the protector of the genetic code (Feroz et al. 2025), is also a tumor suppressor that is capable of regulating cell cycle via triggering G1 phase arrest. When phosphorylated, p53 functions as a transcription factor, increasing the expression of p21, which subsequently causes cell cycle arrest. The activation of the p53/p21 pathway promotes apoptosis through the upregulation of specific proteins. Furthermore, p53 is thought to play a pivotal role in DNA repair mechanisms. Alterations such as dysregulation, inactivation, mutation, or silencing of p53 are associated with various pathologies, including cancer and neurodegenerative pathologies (Kim et al. 2024).

Captivatingly, carotenoids has been demonstrated to decrease the proliferation of numerous cancer cell lines, such as melanoma, lung, prostate, breast, and leukemia, leading to cycle-cycle arrest (Alateyah et al. 2022). A previous study demonstrated that astaxanthin inhibited breast cancer cell growth in experimental animals by inducing p53-mediated cell cycle arrest and programmed cell death (Kim et al. 2024). Moreover, in breast cancer (BC) cell line MDA-MB-231, astaxanthin was demonstrated to hinder cell cycle progression at various phases, such as the G0/G1 and G2/M phases, depending on the context. This arrest is mediated by various mechanisms, including the modulation of tumor suppressor proteins such as p53 and the regulation of downstream factors such as p21. These actions effectively halt cancer cell proliferation, making astaxanthin a promising candidate for therapeutic strategies targeting cancer progression (Alateyah et al. 2022).

##### Suppressing tumor cell migration and invasion

Cancer progression and metastasis involve malignant cell invasion and migration, which require extracellular matrix metalloproteinase remodeling. MMPs, particularly MMP-9, play a critical role in cancer progression by breaking down the extracellular matrix components surrounding cancer cells. This degradation facilitates tumor growth, invasion into nearby connective tissues, and the spread of cancer cells to other parts of the body through the lymphatic system, bloodstream, or direct extension (Herszényi et al. 2012; Cabral-Pacheco et al. 2020). Therefore, one of the usual strategies in the fight against cancer progression is to use matrix metalloproteinase inhibitors or suppress their expression.

Astaxanthin reduces the expression of matrix metalloproteinases 7 and 10 (MMP-7, MMP-10) by suppressing the activation of the PI3K/AKT/mTOR and NF-κB signaling pathways in gastric epithelial cells (Lee et al. 2022). Additionally, in another study, the expression levels of matrix metalloproteinases 1, 2 and 9 in melanoma cell lines (A375 and A20558) decreased significantly after treatment with astaxanthin (Siangcham et al. 2020).

#### Liver cancer

Chronic inflammation accelerates cancer progression by promoting cell growth and tissue changes. Astaxanthin counteracts this by modulating signaling pathways and reducing proinflammatory cytokines, preventing precancerous cells from becoming malignant. It also inhibits the NF-κB signaling, that is crucial for the inflammatory cascade and cancer. NF-κB activity leads to increased proliferation and resistance to cell death (Chang and Xiong 2020). An earlier study stated that astaxanthin returned the concentrations of MDA to normal levels and reduced the levels of caspase-3 and NF-κB in the livers of rats (Bal et al. 2023).

#### Oral cancer

Oral squamous cell carcinoma progression is linked to dysregulated molecular pathways such as the PI3K/Akt signaling, which drives cell proliferation, survival, and tumor progression. Additionally, when persistently activated, NF-κB and STAT-3 stimulate genes that enhance malignancy, including those encoding anti-apoptotic mediators, those involved in angiogenesis, and tissue invasion (Kowshik et al. 2019). Astaxanthin exerts anticancer roles by inhibiting the PI3K/Akt pathway, which in turn reduces the activation of NF-κB and STAT-3 and interferes with feedback mechanisms that sustain cancer cell proliferation and survival. Additionally, a study by Kowishk et al. utilizing a hamster model of oral cancer revealed that consuming dietary astaxanthin downregulates MMP-9 and MMP-2 by regulating the JAK-2/STAT-3 pathway (Kowshik et al. 2014). Moreover, an earlier in vivo study demonstrated that astaxanthin managed to suppress oral carcinomas through promoting apoptosis through the downregulation of Erk/MAPK and PI3K/Akt signaling (Kavitha et al. 2013).

#### Breast cancer

An intriguing aspect of astaxanthin is its selective antiproliferative effects, as some studies indicate that it possesses a greater impact on cancerous cells while sparing normal cells to a greater extent. For example, a 2018 study stated that astaxanthin decreased the migration and proliferation of breast cancer cells (MCF-7) compared with normal cells (McCall et al. 2018). The antiproliferative impact of astaxanthin on the SKBR3 breast cancer cell line have been studied, revealing that astaxanthin hinders cell cycle progression at the G0/G1 phase in a dose-dependent manner. Additionally, it constrains cell proliferation and promotes apoptosis in cancer cells (Hormozi et al. 2019). Moreover, astaxanthin has dual roles in increasing anticancer efficacy and reducing the oxidative stress associated with chemotherapy. An earlier report showed that astaxanthin enhances the anti-proliferative function of carbendazim on MCF-7 cells. It induces G2/M phase cell cycle arrest while mitigating the increased intracellular ROS levels caused by carbendazim (Atalay et al. 2019). Additionally, astaxanthin selectively induces apoptosis, DNA damage, and cell death in breast cancer cells (MDA-MB-231 and T47D), with more pronounced effects in T47D cells. Compared with melatonin, it reduces cell viability and Bcl2 expression at lower doses but has minimal cytotoxic effects on noncancerous MCF10A cells (Karimian et al. 2022). This highlights its ability to act as a targeted anticancer agent with low harm to normal cells.

#### Prostate cancer

Prostate cancer is a prevalent malignancy that is driven by complex mechanisms, including the stimulation of the STAT3 signaling. STAT3 is frequently overexpressed or abnormally activated in prostate cancer, promoting cellular cascades including proliferation, survival, invasion, and metastasis. Such cascade is associated with the upregulation of genes such as cyclins, which are critical cell cycle regulators, in addition to MMPs, and the angiogenic VEGF. Astaxanthin exerts anticancer effects on aggressive PCa DU145 cells by inhibiting STAT3. It suppresses cell proliferation and colony formation, especially when combined with STAT3 silencing. Astaxanthin induces apoptosis by upregulating pro-apoptotic mediators while downregulating the anti-apoptotic ones. Additionally, it inhibits cell migration and invasion by reducing the expression of MMPs and VEGF, which are crucial for metastasis (Sun et al. 2020).

#### Colon cancer

Colon cancer arises from a wide array of genetic, environmental, and dietary factors driven by inflammatory and oncogenic pathways. The key contributors include the NF-κB (which promotes inflammation and tumor progression), COX-2 (which supports angiogenesis and inhibits apoptosis), MMP, and ERK/Akt pathways (which enhance cell survival and resistance to apoptosis). These disruptions collectively fuel tumor growth, invasion, and metastasis. Astaxanthin shows strong chemopreventive and therapeutic effects against experimental rat models of colon cancer by targeting several key pathways. It reduces inflammation by reducing NF-κB signaling and COX-2, suppresses tumor invasion by downregulating MMP-2 and MMP-9, and promotes apoptosis through increased annexin-V and caspase-3 activity. Additionally, it decreases mast cell infiltration, decreasing their role in tumor growth and invasion (Nagendraprabhu and Sudhandiran 2011). The anticancer activities of astaxanthin are summarized in Table 1.

**Table 1:** The anticancer activities and mechanisms of action of astaxanthin

Cancer type	Type of study	Mechanism of action	References
Liver cancer	In vivo	Reduces chronic inflammation slowing cancer progression and promotion of cell growth and tissue changes by: Modulating several signaling pathways and reduces pro-inflammatory cytokines Inhibiting NF-κB signaling Reducing caspase-3 and NF-κB levels in rat liver	Bal et al. (2023)
Oral cancer	In vivo	Blocks PI3K/Akt signaling pathway, reducing NF-κB and STAT-3 activation Inhibits MMP-9 and MMP-2 expression via JAK-2/STAT-3 pathway Induces apoptosis through Erk/MAPK and PI3K/Akt signaling inhibition	Kavitha et al. (2013), Kowshik et al. (2014)
Breast cancer	In vitro	Constrains proliferation and migration of MCF-7 Induces G0/G1 phase cell cycle arrest Enhances anti-proliferative effects of carbendazim Selectively enhances apoptosis in cancer cells	McCall et al. (2018), Atalay et al. (2019), Hormozi et al. (2019), Karimian et al. (2022)
Prostate cancer	In vitro	Inhibits STAT3 signaling pathway Suppresses cell proliferation and colonization Induces apoptosis by upregulating Bax, caspase-3 and caspase-9 Reduces MMPs and VEGF expression	Sun et al. (2020)
Colon cancer	In vivo	Reduces inflammation by inhibiting NF-κB and COX-2 Suppresses tumor invasion by downregulating MMP-2 and MMP-9 Promotes apoptosis through increased annexin-V and caspase-3 activity	Nagendraprabhu and Sudhandiran (2011)
General cancers	In vitro	Induces apoptosis via mitochondrial pathway Inhibits uncontrolled cell proliferation by inducing p53-mediated cell cycle arrest Suppresses tumor cell migration and invasion by reducing MMP expression	Alateyah et al. (2022), Lee et al. (2022)
Indirect effects	In vivo and in vitro	Antioxidant activity reduces oxidative stress Anti-inflammatory properties reduce chronic inflammation, which promotes tumor growth Modulates NF-κB, Nrf2, and APA-1 signaling pathways	Davinelli et al. (2022)

### Nephroprotective effects

Astaxanthin has earned recognition as a potent antioxidant, showing significant potential in preventing kidney cell damage. By engaging multiple pathways, astaxanthin provides protection against renal injuries caused by various factors.

#### Diabetes-induced nephrotoxicity

Diabetic nephropathy is a chief factor driving the progression of diabetes to end-stage renal disease, with podocyte damage being a key indicator of disease progression. Autophagy, which helps maintain cellular homeostasis by degrading damaged proteins and organelles, is vital for preventing posttranslational damage. However, high glucose levels inhibit autophagy in podocytes, worsening kidney damage and accelerating diabetic nephropathy. Astaxanthin may protect against diabetes-induced kidney injury in rats by increasing autophagic activity in podocytes, potentially slowing disease progression (Hong et al. 2024). Hyperglycemia contributes significantly to damage mediated by ROS. In diabetic rats, astaxanthin administration demonstrated hypoglycemic and antihyperglycemic effects by lowering fasting blood glucose levels. Its antidiabetic activity is mainly associated with its antioxidant properties, particularly through its aptitude to scavenge free radicals and suppress lipid peroxidation, which support the survival of pancreatic β-cells (Sila et al. 2015).

#### Burn-induced nephrotoxicity

In burn-induced nephrotoxicity, kidneys exhibit tubular epithelial necrosis and dilation, driven by increased ROS generation, reduced antioxidant enzyme activity, and elevated inflammatory mediators. MyD88-dependent TLR4/NF-κB signaling is fundamentally involved in controlling inflammation related to burn-induced acute renal injury. Heme oxygenase-1 (HO-1), an endogenous antioxidant, offers nephroprotection by mitigating oxidative damage and regulating biological processes. Astaxanthin treatment of severely burned rats reduced the levels of inflammatory mediators, inhibited the TLR4/MyD88/NF-κB axis, and upregulated HO-1 expression in a dose-dependent manner, aiding in oxidative stress regulation and TLR4 pathway modulation (Guo et al. 2021).

The PI3K/Akt signaling contributes significantly to counteracting renal tubular apoptosis following burn injury. Bad, a proapoptotic protein of the Bcl-2 family, interacts with mitochondrial Bcl-xL, leading to the mitochondrial release of cytochrome c and caspase activation, which indicates early mitochondrial injury and apoptosis in acute kidney damage. Activated Akt (p-Akt) phosphorylates Bad and Bax, inhibiting their proapoptotic functions and reducing downstream protein expression. In vivo, astaxanthin modulated the PI3K/Akt pathway, increasing Bad phosphorylation and suppressing cytochrome c expression, thereby mitigating burn-induced nephrotoxicity (Guo et al. 2015).

#### Hyperuricemia-induced nephrotoxicity:

Abnormal uric acid (UA) synthesis in the liver is a significant contributor to elevated UA levels in the bloodstream. Within the purine nucleotide metabolic pathway, adenosine deaminase (ADA) and xanthine oxidase (XOD) serve as critical enzymes facilitating the conversion of purines to UA. Excessive accumulation of UA often induces mesangial cells to produce substantial amounts of ROS, activates the NLRP3 inflammasome, and induces the production and release of pro-inflammatory mediators through the NF-κB pathway. The NF-κB signaling pathway is essential for regulating the release of inflammatory mediators, while the NLRP3 inflammasome, which consists of NLRP3, ASC, and caspase-1, activates caspase-1 and enhances the production of inflammatory mediators. Together, the NLRP3 and NF-κB pathways are central to kidney inflammation. Astaxanthin alleviates hyperuricemia and kidney inflammation induced by potassium oxonate and hypoxanthine in mice by suppressing the activities of XOD and ADA, while inhibiting the NF-κB and NLRP3 signaling pathways (Zhuang et al. 2021).

#### Unilateral ureteral obstruction (UUO)-induced nephrotoxicity

Unilateral ureteral obstruction (UUO) leads to the loss of peritubular capillaries, a condition that has been identified as a key contributor to renal interstitial fibrosis. This fibrosis represents a key pathological hallmark of chronic renal disorders. Promoting the repair of impaired capillary networks is crucial for reducing interstitial fibrosis, where angiogenesis serves as a key mechanism in the restoration process. VEGF is indispensable for inducing endothelial cells to form tube-like structures and serves as a key mediator of angiogenesis. Conversely, thrombospondin-1 (TSP-1), an antiangiogenic molecule, can exacerbate progressive renal disease. TSP-1 acts as an endogenous activator of TGF-β1, a pivotal mediator involved in the development of renal interstitial fibrosis. Astaxanthin mitigated renal interstitial fibrosis induced by UUO in mice by increasing the peritubular capillary density through the upregulation of VEGF-A and the downregulation of TSP-1. Furthermore, astaxanthin has antifibrotic effects by suppressing TGF-β1/Smad pathway activation (Zhao et al. 2019).

#### Contrast agent-induced acute kidney injury (CI-AKI)

Contrast agent-induced acute kidney injury (CI-AKI) arises from a complex interplay of oxidative stress, inflammation, and consequently apoptosis within renal tubular epithelial cells (Gao and Li 2018). Central to this process are ROS, which drive inflammatory mechanisms by either directly or indirectly enhancing the productions of pro-inflammatory mediators. Furthermore, ROS act as key activators of the NLRP3 inflammasome, causing the production and release of IL-1β and promoting apoptotic cell death, thus exacerbating renal damage. Astaxanthin alleviates iohexol-induced injury in human proximal renal tubular epithelial cells by reducing ROS production and inhibiting NLRP3 inflammasome, along with its downstream apoptotic effects (Gao et al. 2019).

Sirtuin 2 (Sir2), a member of the 3rd class of histone deacetylases that depend on nicotinamide adenine dinucleotide (NAD +), is a main modulator of various molecular functions (Dali-Youcef et al. 2007). SIRT1, a homolog of Sir2, plays a critical role in modulating glucose and lipid metabolic cascades, controlling the cell cycle, and mitigating both physiological and pathological processes related to inflammation and oxidative stress (Liu et al. 2018a, b). Additionally, FOXO3a is essential for cell survival, primarily through its ability to transactivate antioxidant enzymes which protects cells from oxidative damage (Ali et al. 2021).

Following the administration of iohexol, there was an upregulation in SIRT1 expression and a reduction in FOXO3a expression. However, astaxanthin exerts renoprotective effects both in vivo and in vitro by modulating the SIRT1/FOXO3a signaling pathway. At higher doses, astaxanthin downregulates SIRT1 while upregulating FOXO3a, leading to a decrease in oxidative stress and a decline in the apoptosis of renal tubular epithelial cells induced by CI-AKI (Liu et al. 2018a, b).

#### Trivalent inorganic arsenic-induced renal injury

Inorganic arsenic (iAs), a toxic environmental metalloid, targets the kidneys as a critical organ for trivalent iAs toxicity. As₂O₃ exacerbates oxidative stress by increasing ROS production and decreasing the mitochondrial membrane potential. Astaxanthin counteracts such effects by restoring antioxidant enzyme levels, preserving mitochondrial function in a reduced state, and ameliorating oxidative stress-induced renal damage triggered by As₂O₃ in Wistar rats (Wang et al. 2014).

#### Drug-induced nephrotoxicity

Several in vivo studies have demonstrated that astaxanthin can effectively ameliorate nephrotoxicity induced by various drugs, including vancomycin, gentamicin, cisplatin, lithium, and colistin (Ghlissi et al. 2014; Akca et al. 2018; Shi et al. 2021; Liu et al. 2023; Erbaş et al. 2024). Most of these drugs induce nephrotoxicity due to an imbalance between oxidation and the antioxidant system, leading to increased oxidative stress, inflammation, and apoptosis in kidney tissue. Astaxanthin has antioxidant and anti-inflammatory properties through multiple pathways. As highlighted in a previous study on acute kidney injury induced by gentamicin, astaxanthin activates the antioxidant defense pathways of Nrf2 and HIF-α while also suppressing the MAPK/ERK signaling pathway (Liu et al. 2023). Additionally, astaxanthin reduces apoptosis, as observed in cisplatin-induced kidney injury in rats, by decreasing caspase-3 expression in renal tubular epithelial cells exposed to nephrotoxic drugs (Akca et al. 2018).

Astaxanthin exhibits promising nephroprotective potential by reducing oxidative injury, inflammation, and fibrosis in experimental kidney injury. Nevertheless, variations in animal species, disease models, and treatment duration limit the comparability of outcomes. Clinical data are still insufficient, and future studies should investigate pharmacokinetic correlations and optimal therapeutic windows for renal protection.

### Antidiabetic effects

Numerous investigations have demonstrated that astaxanthin has beneficial effects on diabetes via mitigating insulin resistance by safeguarding myocytes from oxidative damage induced by hyperglycemia in pancreatic β-cells (Uchiyama et al. 2002). Astaxanthin (15, 30, 50 mg/kg) markedly elevated adiponectin and the expression of its receptors AdipoR1 and AdipoR2, resulting in increased HDL-C levels and greater insulin sensitivity. Furthermore, it elevated the expression of PPARγ and UCP2 in rats with STZ-induced insulin resistance (Zhuge et al. 2021). Astaxanthin administration (5 and 10 mg/kg) for one week reduced plasma glucose levels in alloxan-induced diabetic rats (Wang et al. 2012a, b). Similarly, astaxanthin dramatically decreased blood glucose levels (Zhang and Xu 2018a, b). Moreover, in *Mus musculus* pancreatic β-cell line (βTC-tet), astaxanthin reportedly safeguards β-cells by blocking the release of proinflammatory cytokines and maintaining the capacity of islet cells to generate insulin, but no substantial variation in β-cell mass was noted (Sakayanathan et al. 2024). Moreover, astaxanthin reduced the production of ROS, including O2–, NO, and ONOO^–^, in a dose-dependent manner in proximal tubular epithelial cells exposed to high glucose. Similarly, the hyperglycemia-induced generation of O_2_^–^ is inhibited in the retinas of db/db mice. Mice, U937 cells, vascular endothelial cells, glomerular mesangial cells, and hepatic cells from STZ-induced diabetic rats were treated with astaxanthin (Dong et al. 2013; Park et al. 2015; Chen et al. 2018).

Astaxanthin has been shown to rectify redox imbalance in diverse tissues and cells by increasing the endogenous antioxidant enzymes and compounds (Li et al. 2016). Moreover, astaxanthin inhibits the expression of collagen, fibronectin, and TGF-β1, all of which are linked to glomerular matrix overproduction and glomerulosclerosis, while concurrently increasing the levels of connexin43, a transmembrane gap junction protein whose diminished production, as observed in diabetes, correlates with the senescence of glomerular mesangial cells and ultimately nephropathy in the kidneys of db/db mice (Chen et al. 2018). The augmented production of connexin43 subsequently stimulates the Nrf2/ARE pathway, a crucial modulator of oxidative stress, thereby alleviating oxidative stress-induced kidney damage in rats. A reduction in oxidative stress biomarkers and a simultaneous increase in the antioxidant systems of the kidneys are noted following astaxanthin administration (Zhu et al. 2018).

### Dyslipidemia and metabolic syndrome

Astaxanthin managed to decrease dyslipidemia and alleviate metabolic syndrome in experimental models. Moreover, in apoE-knockout animals subjected to a high-fat and cholesterol diet, astaxanthin upregulated the LDL-receptor (LDLR), 3-hydroxy-3-methylglutaryl CoA (HMG-CoA) reductase, and sterol regulatory element binding protein 2 (SREBP-2) in the liver, potentially accounting for the evident hypocholesterolemic function of astaxanthin (Aoi et al. 2008). Similarly, the mRNA expression levels of carnitine palmitoyl transferase 1 (CPT1), acetyl-CoA carboxylase β (ACACB), and acyl-CoA oxidase (ACOX) were markedly elevated by astaxanthin supplementation, indicating that the triglyceride-lowering impact of astaxanthin may be attributed to increased hepatic β-oxidation. In addition, astaxanthin influences critical components of cholesterol efflux from macrophages by increasing the expression of ATP-binding cassette transporters (ABCs) A1 and G1, thus facilitating cholesterol efflux (Yang et al. 2011). Astaxanthin reduces plasma triglyceride and upregulates antioxidant genes regulated by Nrf2 in diet-induced obese mice (Yang et al. 2014). Furthermore, astaxanthin stabilized atherosclerotic plaques in hyperlipidemic rabbits and reduced macrophage infiltration and vascular cell death (Li et al. 2004).

Numerous reports have demonstrated that alterations in the intracellular redox balance can influence lipid metabolism. Increased oxidative stress is correlated with lipid buildup in adipose tissue that influences the control of hepatic lipid biosynthesis (Napolitano et al. 2001; Furukawa et al. 2017). During exercise, the formation of ROS in skeletal muscle escalates in tandem with increased energy expenditure, potentially influencing the consumption of energy substrates in muscle and resulting in lipid metabolism disorders. It has been previously reported that astaxanthin enhanced lipid use during exercise, resulting in increased endurance and effective body fat reduction with training in mice (Aoi et al. 2008). The rise in fatty acyl-CoA transport into mitochondria through CPT1 during exercise may contribute to lipid metabolism facilitated by the antioxidant properties of astaxanthin. Astaxanthin is anticipated to enhance aerobic performance and regulate body weight through alterations in muscle energy metabolism due to its antioxidant properties (Aoi et al. 2008).

The alterations in the lipid profile following astaxanthin supplementation in an in vitro membrane model were comparable to those of lutein and β-carotene. The results indicated that astaxanthin maintained membrane integrity by preventing lipid peroxide generation, while lutein and β-carotene not only compromised the membrane structure but also elevated lipid hydroperoxide levels (O'Connor and O’Brien 1998). Astaxanthin functions as an agonist for PPAR-γ and PPAR α, thereby diminishing cellular lipid accumulation in lipid-laden hepatocytes (Jia et al. 2012). During the initiation of mitochondrial aerobic metabolism, astaxanthin enhances the uptake of peroxisome proliferator-activated receptor-γ coactivator 1-α (PGC-1α) and promotes lipid utilization in skeletal muscle (Liu et al. 2014). A separate 12-week trial included 61 nonobese Japanese participants with moderate hypertriglyceridemia showed that doses of 12 and 18 mg/day astaxanthin managed to reduce serum triglyceride levels, whereas doses of 6 and 12 mg/day elevated serum HDL cholesterol (Yoshida et al. 2010).

### Ocular health

Astaxanthin has demonstrated significant potential for improving eye health by targeting oxidative stress and inflammation, which are critical factors in a variety of ocular diseases. Studies have shown that astaxanthin enhances outcomes in conditions that affect both the anterior and posterior segments of the eye, including diabetic retinopathy, age-related macular degeneration (AMD), and dry eye syndrome (Giannaccare et al. 2020). This is accomplished by minimizing oxidative stress-induced retinal damage and improving blood flow in ocular tissues, which is essential for the preservation of visual acuity and retinal health (Sekikawa et al. 2023).

Mechanistically, astaxanthin accumulates in ocular tissues, where it regulates inflammatory pathways and reduces cellular stress. For example, in dry eye syndrome, astaxanthin reduces the production of proinflammatory cytokines, which promotes tear film stability and alleviates symptoms (Tian et al. 2022). The antioxidative properties of astaxanthin in AMD safeguard the retinal pigment epithelium and photoreceptor cells from lipid peroxidation, a process that is further exacerbated by extended light exposure and aging. Furthermore, its ability to improve mitochondrial function is essential for the maintenance of visual performance, as it contributes to improved energy production in retinal cells (Yang and Wang 2022).

The advantages of astaxanthin supplementation for visual function in healthy individuals have also been emphasized in RCTs. Significant enhancements in visual acuity and contrast sensitivity were reported in a six-week supplementation study, which were likely the result of reduced oxidative stress and enhanced retinal resilience (Sekikawa et al. 2023). Such results highlight the ability of astaxanthin to be utilized as a therapeutic agent for managing and preventing eye disorders, suggesting that it is a natural, well-tolerated option for ocular health maintenance.

In summary, astaxanthin demonstrates beneficial effects on visual function and retinal protection through antioxidant defense enhancement and microvascular improvement. Yet, the evidence base is still limited by small sample sizes and short intervention periods. Future research should prioritize larger controlled trials and pharmacodynamic assessments of ocular delivery formulations.

### Auditory health

Astaxanthin has potential advantages for auditory health by alleviating oxidative stress and inflammation, which are significant factors in hearing impairment. In diabetic rat models, it significantly decreases hearing loss by neutralizing ROS and augmenting antioxidant defenses (Toprak and Dedeoğlu 2022). In vitro*,* astaxanthin also protects against ototoxicity induced by cisplatin, a chemical agent known to induce hearing loss, by preventing cochlear apoptosis and reducing oxidative damage, with enhanced effects when encapsulated in ROS-responsive nanoparticles (Gu et al. 2022). Similar protective mechanisms were reported in rat studies where astaxanthin supplementation improved auditory thresholds and reduced cochlear damage caused by cisplatin exposure (Kınal et al. 2021). Additionally, astaxanthin-loaded nanoparticles have shown potential in treating secretory otitis media by reducing inflammation and enhancing drug delivery (Yang et al. 2024).

### Female health

Astaxanthin has demonstrated promising potential in improving female reproductive health, with a particular emphasis on the management of polycystic ovary syndrome (PCOS), improving the quality of assisted reproductive technology (ART) outcomes, and increasing fertility parameters. In PCOS, ovarian function is significantly influenced by inflammation and oxidative stress. As evidenced by the reduced proinflammatory cytokines and improved insulin resistance and hormone balance, the potent antioxidant and anti-inflammatory properties of astaxanthin mitigate such effects, resulting in improved ovarian responses and higher-quality embryos. Additionally, the administration of 6 mg astaxanthin per day for 2 months in RCTs resulted in a reduction in inflammation, which in turn significantly improved the outcomes of ART for women with PCOS (Fereidouni et al. 2024; Fu et al. 2024).

In the context of patients undergoing ART, astaxanthin enhances reproductive outcomes by reducing oxidative stress, which is crucial for improving embryo quality and implantation rates. Studies indicate that astaxanthin supplementation supports oocyte function and alleviates ER stress in ovarian cells, which are key to maintaining cellular homeostasis during ART procedures. Additionally, its antioxidative effects decrease apoptosis in ovarian follicles, thereby improving the ovarian reserve and response in poor ovarian responders undergoing ART (Rostami et al. 2023; Shafie et al. 2024).

Astaxanthin also benefits overall fertility by promoting hormonal balance and reducing systemic oxidative stress, which are essential factors in reproductive success. For example, in a RCT containing 56 patients with PCOS, astaxanthin treatment improved pregnancy rates by increasing embryo quality and reducing the levels of inflammatory markers (Jabarpour et al. 2024a, b). Such findings underscore the value of astaxanthin as a dietary astaxanthin supplementation in mitigating female reproductive-related disorders, especially in situations exacerbated by oxidative stress and inflammation.

Experimental studies indicate that astaxanthin improves hormonal balance, and ovarian function via oxidative stress attenuation. Nonetheless, the applicability of such pre-clinical reports to human reproductive health remains uncertain. Well-designed human trials are essential to confirm dose–response relationships and mechanism-specific benefits.

## Clinical evidence and safety considerations of Astaxanthin

Although numerous preclinical studies have demonstrated antioxidant, anti-inflammatory, and neuroprotective effects of astaxanthin, clinical data in humans remain limited and often heterogeneous in design, sample size, and dosage. Available studies have primarily focused on metabolic, cardiovascular, visual, and reproductive outcomes, with promising results. Most of them are often focused on biomarkers or intermediate outcomes rather than hard clinical endpoints (Table 2).

**Table 2:** Clinical studies on astaxanthin in humans and their main outcomes

Disease or condition	Study design	Number of participants	Dose and duration	Outcomes	Adverse effects	References
Arterial stiffness in renal transplant recipients	Randomized, placebo-controlled trial	66 renal transplant recipients	12 mg/day astaxanthin (BioAstin) for one-year	Primary outcomes included 1) arterial stiffness measured by aortic pulse wave velocity (PWV), 2) oxidative stress assessed by plasma isoprostanes and 3) inflammation by plasma pentraxin 3 Secondary outcomes included changes in vascular function assessed using the brachial artery reactivity technique, carotid artery intimal medial thickness, augmentation index, left ventricular afterload and additional measures of oxidative stress and inflammation	No serious adverse events were reported	Fassett et al. (2008a, b)
Coronary artery disease (CAD)	Randomized, double-blind, placebo-controlled trial	50 CAD patients	12 mg/day astaxanthin for 8 weeks	Body composition, glycemic indices, serum levels of TNF-α, Sirtuin1, TG and HDL-C did not differ substantially A significant reduction in total cholesterol (− 14.95 ± 33.57 mg/dl, *p* < 0.05) and LDL-C (− 14.64 ± 28.27 mg/dl, *p* < 0.05) was reported	Participants did not report any side effects	Heidari et al. (2023)
Type 2 diabetes mellitus	Randomized, placebo-controlled trial	44 type 2 diabetic patients	8 mg/day astaxanthin for 8 weeks	Astaxanthin increased the serum adiponectin concentration and reduced visceral body fat mass (*P* < 0.01), serum triglyceride and very-low-density lipoprotein cholesterol concentrations, and systolic blood pressure (*P* < 0.05) Astaxanthin reduced the fructosamine concentration (*P* < 0.05) and marginally reduced the plasma glucose concentration (*P* = 0.057)	No adverse effects were reported	Mashhadi et al. (2018)
	Randomized, double-blind placebo-controlled trial	50 T2DM subjects	10 mg/day astaxanthin + metformin for 12 weeks	A significant increase in SOD and catalase activities, as well as Nrf2 protein expression were reported No significant changes in serum malondialdehyde and TAC were observed A significant increase was observed in TAC (32.67 ± 6.73) compared to before supplementation (25.86 ± 5.98)	Astaxanthin was well tolerated, and no patient reported any significant adverse effects	Rad et al. (2022)
Heart failure	Prospective pilot study	16 heart failure patients with left ventricular dysfunction	3-month supplementation reported in at least one pilot	Serum Diacron reactive oxygen metabolite (dROM) level decreased from 385.6 ± 82.6 U.CARR to 346.5 ± 56.9 U.CARR (*P* = 0.041) despite no changes in biological antioxidant potential and urinary 8-hydroxy-2′-deoxyguanosine levels Left ventricular ejection fraction (LVEF) increased from 34.1 ± 8.6% to 38.0 ± 10.0% (*P* = 0.031) and 6-min walk distance increased from 393.4 ± 95.9 m to 432.8 ± 93.3 m (*P* = 0.023) Significant relationships were observed between percent changes in dROM level and those in LVEF Astaxanthin suppressed oxidative stress and improved cardiac contractility and exercise tolerance	No complaints or adverse events related to astaxanthin administration were observed	Kato et al. (2020a, b)
	A sub-study of the prospective observational pilot study	17 patients with heart failure	12 mg of astaxanthin with 40 mg of tocotrienol and 30 mg of L-ascorbic acid 2-glucoside for 3 months	The specific activity scale score increased from the median of 4.5 (interquartile range, 2.0) to 6.5 (interquartile range, 1.1) metabolic equivalent (*P* = 0.001), and the physical and mental component summary scores increased from 46.1 ± 9.2 to 50.8 ± 6.8 (*P* = 0.015) and from 48.9 ± 9.1 to 53.8 ± 4.8 (*P* = 0.022), respectively	Not reported	Ishiwata et al. (2021)
Visual function	Randomized, placebo-controlled study	60 healthy adults	A diet containing 9 mg/day astaxanthin for 6 weeks	Participants aged ≥ 40 years showed less screen-related eye fatigue and better maintained vision clarity (*p* < 0.05) In participants aged < 40 years, no significant difference was reported No significant difference was found in functional visual acuity and pupil constriction rate	Not reported	Sekikawa a et al. (2023)
Polycystic ovary syndrome (PCOS)	Triple-blind randomized trial	58 infertile women with diagnosed PCOS	6 mg astaxanthin twice daily for 8 weeks	A significant reduction was observed in fasting blood sugar, HOMA-IR, fasting insulin, MDA, low-density lipoprotein-cholesterol, and TC/HDL-C ASX significantly increased TAC, HDL-C, and quantitative insulin sensitivity check index (QUICKI)	Astaxanthin was reported to be safe	Jabarpour et al. (2024a, b)
	Non-randomized comparative observational study	92 PCOS patients undergoing in vitro fertilization/intracytoplasmic sperm injection (IVF/ICSI)	Astaxanthin compound nutrient (ACN) complementary therapy for 3 months prior to IVF/ICSI	There were no significant differences in the patient’s duration of stimulation, total gonadotropin dose, peak E2 levels, and number of retrieved oocytes The number of 2 pronucleus fertilization, transferable embryos, and high-quality embryos was significantly higher in the ACN group For both fresh and frozen embryo transplantation, positive pregnancy outcomes increased in PCOS patients who received supplementation of ACN for 3 months BMI, anti-Müllerian hormone, fasting insulin, HOME-IR, basal luteinising hormone (bLH), and basal testosterone (bT) decreased compared to before ACN treatment	Not reported	Fu et al. (2024)
	Randomized, triple-blind, placebo-controlled trial	44 infertile PCOS patients	6 mg/day astaxanthin for 8 weeks	A significant decrease in IL-6 and IL-1β (*P* = < 0.01 for both) was reported Follicular fluid cytokine levels and oxidative stress markers did not differ significantly Reproductive outcomes, including the number of oocytes retrieved (*P* = 0.01), the MII oocyte count (*P* = 0.007), oocyte maturity rate (*P* = 0.02) and number of frozen embryos (*P* = 0.03) significantly improved after intervention No significant differences were found in chemical, clinical and multiple pregnancies between the groups	Not reported	Fereidouni et al. (2024)
	Randomized, triple-blind, placebo-controlled trial	60 infertile patients with poor ovarian response (POR) from POSEIDON Group 4 (the poorest prognosis category, age > 35 and poor ovarian reserve (anti-müllerian hormone < 1.2 ng/ml or antral follicle count < 5) undergoing intracytoplasmic sperm injection	12 mg/day astaxanthin for 8 weeks	A significant elevation in TAC (*P* = 0.030), accompanied by a significant reduction in serum MDA (*P* = 0.005) was reported No significant differences were observed in oxidative stress markers in follicular fluid Significant reductions in the serum IL-6 (*P* < 0.001), IL-8 (*P* = 0.001), and VEGF (*P* = 0.002) levels were shown FF levels of IL-6 (*P* = 0 < 001), IL-8 (*P* = 0.036), VEGF (*P* = 0.006), and cfDNA (*P* < 0.001) were significantly lower Significant increases in the number of retrieved oocytes (*P* = 0.003), MII oocytes (*P* = 0.004), frozen embryos (*P* = 0.037), and high-quality embryos (*P* = 0.014) were reported	No adverse effects or instances of toxicity were reported throughout the intervention	Shafie et al. (2024)
Endometriosis	Randomized, triple-blind, placebo-controlled trial	50 infertile women with endometriosis candidate for assisted reproductive techniques	6 mg/day astaxanthin for 12 weeks	Increased serum levels of TAC (*P* = 0.004) and SOD (*P* = 0.010) were observed Serum MDA (*P* = 0.031) decreased significantly Significantly lower serum levels of IL-1β (*P* = 0.000), IL-6 (*P* = 0.024) and TNF-α (*P* = 0.038) were observed Improved the number of oocytes retrieved (*P* = 0.043), number of mature oocytes (*P* = 0.041), and high-quality embryos (*P* = 0.024)	The patients reported no adverse effects or toxicity during the intervention	Rostami et al. (2023)

### Safety profile of astaxanthin

Astaxanthin is widely regarded as harmless and well-tolerated, with no significant adverse effects reported in either preclinical or clinical studies. Natural astaxanthin has been evaluated in several clinical trials, without indications of toxicity or liver damage (Brendler and Williamson 2019). The only minor adverse effect noted at high doses is reddening of the stool. Regulatory assessments confirm that astaxanthin supplementation at doses up to 12 mg/day poses no safety concerns. Acute toxicity studies in animals further support this safety profile, as the LD_50_ and the lack of observed adverse effects for astaxanthin-rich biomass exceed 12,000 mg/kg and 465 mg/kg/day, respectively, in rats (Satoh 2016). However, while synthetic astaxanthin has shown species-specific effects at extremely high doses (200–1000 mg/kg/day in rats), its safety in humans remains unverified, necessitating further clinical trials before its approval for human consumption (Brendler and Williamson 2019).

While interpreting and extrapolating the findings reported in this review, it is important to consider the strengths and limitations of the different study models used. In vitro reports provide valuable mechanistic insights and allow a controlled evaluation of molecular pathways; however, they lack the complexity of whole-organism physiology. Meanwhile, in vivo animal studies can reflect systemic and tissue-specific responses more accurately, however the species differences may limit the direct extrapolation to humans. Finally, clinical studies offer the highest level of relevance both physiologically and therapeutically, although they are often limited by sample size and subjects’ heterogeneity in terms of ethnic origin and environment.

Nonetheless, explicit safety reporting is often sparse, and standardized data on optimal dosing, formulation, pharmacokinetics, drug–nutrient interactions, and long-term safety in patients with comorbidities remain lacking. Therefore, larger, well-powered randomized controlled trials with pre-specified safety endpoints and standardized dosing/formulations are required before firm clinical recommendations can be made. Therefore, integrating evidence across these complementary approaches is essential to accurately assess astaxanthin’s therapeutic potential and guide future translational research.

## Delivery systems for astaxanthin: enhancing bioavailability and efficacy

The therapeutic potential of astaxanthin is often limited by its poor water solubility, low bioavailability, and susceptibility to degradation under environmental conditions of light, heat, and oxygen (Zhang et al. 2025). To overcome these challenges, advanced delivery systems have been employed to enhance drug stability, bioavailability, and targeted delivery. Liposomal formulations and ROS-responsive nanoparticle encapsulation have shown promise in improving astaxanthin solubility and cellular uptake, particularly in applications such as hepatoprotection and ototoxicity (Chiu et al. 2016; Gu et al. 2022). Similarly, nanoparticle-based delivery systems, including polymeric nanoparticles and solid lipid nanoparticles, have been employed to achieve controlled release and improve the efficacy of astaxanthin in various diseases, including ocular diseases (Sorasitthiyanukarn et al. 2022; Sun et al. 2023; Yang et al. 2024). Encapsulation techniques, such as freeze drying, spray drying, and coacervation, have also been utilized to protect astaxanthin from degradation and enhance its incorporation into functional foods and nutraceuticals (Martínez-Álvarez et al. 2020; Panagiotakopoulos and Nasopoulou 2024).

Despite the extensive evidence supporting astaxanthin’s biological activity, the poor bioavailability represents a major challenge to clinical translation. Such recent advances in delivery systems have shown promise in enhancing stability and bioavailability. Nevertheless, few clinical studies have evaluated these formulations directly. Future research should focus on comparative pharmacokinetic analyses, dose optimization, and exploring synergistic combinations to improve its therapeutic efficacy and consistency across populations.

## Future prospects and challenges in astaxanthin research

In fact, several challenges hamper the widespread application of astaxanthin, in spite of its promising therapeutic potential. Key issues include its low bioavailability, instability in formulation, and high cost of production from natural sources such as *Hematococcus pluvialis* (Li et al. 2020a, b). To address these challenges, future reports ought to emphasize on synthetic biology approaches to produce astaxanthin more efficiently and cost-effectively, besides the development of novel biomaterials for improved delivery (Fang et al. 2019; Zhang et al. 2025). Additionally, large-scale clinical trials are needed to corroborate the efficacy and safety of astaxanthin in humans, particularly in emerging areas such as neurodegenerative diseases, metabolic syndrome, and female reproductive health (Fakhri et al. 2019; Shafie et al. 2024). Interdisciplinary collaboration between researchers, clinicians, and industry stakeholders will be crucial to overcoming regulatory hurdles and scaling up production. In the future, astaxanthin holds immense potential for integration into personalized medicine and combination therapies, where its beneficial properties can be synergistically combined with other therapeutic agents (Rad et al. 2022; Golestani et al. 2024; Zhang et al. 2024; Copat et al. 2025). By addressing these challenges and exploring new applications, astaxanthin can be positioned as a cornerstone of next-generation therapeutics and nutraceuticals.

## Conclusion

Our review suggests that astaxanthin may have biological activity in both in vitro and in vivo models. Such investigations highlight the role of astaxanthin and its potential benefits as a cardioprotective, antioxidant, anti-inflammatory, anticancer, antidiabetic, immunostimulant, and neuroprotective drug. Astaxanthin is utilized in a wide array of commercial applications. The present review provides up-to-date information on astaxanthin chemistry, biological activities, and health benefits. Future reports ought to underscore the impact of astaxanthin esters on diverse biomedical functions, as well as their utility in nutraceutical and medicinal applications. Further research into their metabolic pathways, as well as molecular mechanistic studies is needed before they can be used commercially. Finally, these observations highlight astaxanthin’s pleiotropic biological effects and its potential as a multi-target therapeutic compound, while emphasizing the need for methodological consistency and optimized delivery strategies to maximize its clinical benefits.

## Data Availability

All the data are available within the manuscript.
